# Assessing the genetic background and genomic relatedness of red cattle populations originating from Northern Europe

**DOI:** 10.1186/s12711-021-00613-6

**Published:** 2021-03-06

**Authors:** Christin Schmidtmann, Anna Schönherz, Bernt Guldbrandtsen, Jovana Marjanovic, Mario Calus, Dirk Hinrichs, Georg Thaller

**Affiliations:** 1 Institute of Animal Breeding and Husbandry, Christian-Albrechts-University Kiel, 24098 Kiel, Germany; 2 Department of Molecular Biology and Genetics, Center for Quantitative Genetics and Genomics, Aarhus University, 8830 Tjele, Denmark; 3 Present Address: Department of Animal Science, Aarhus University, 8830 Tjele, Denmark; 4 Present Address: Department of Animal Sciences, Department of Animal Breeding and Husbandry, University of Bonn, 53115 Bonn, Germany; 5 Animal Breeding and Genomics, Wageningen University and Research, 6700AH Wageningen, The Netherlands; 6 Department of Animal Breeding, University of Kassel, 37213 Witzenhausen, Germany

## Abstract

**Background:**

Local cattle breeds need special attention, as they are valuable reservoirs of genetic diversity. Appropriate breeding decisions and adequate genomic management of numerically smaller populations are required for their conservation. At this point, the analysis of dense genome-wide marker arrays provides encompassing insights into the genomic constitution of livestock populations. We have analyzed the genetic characterization of ten cattle breeds originating from Germany, The Netherlands and Denmark belonging to the group of red dairy breeds in Northern Europe. The results are intended to provide initial evidence on whether joint genomic breeding strategies of these populations will be successful.

**Results:**

Traditional Danish Red and Groningen White-Headed were the most genetically differentiated breeds and their populations showed the highest levels of inbreeding. In contrast, close genetic relationships and shared ancestry were observed for the populations of German Red and White Dual-Purpose, Dutch Meuse-Rhine-Yssel, and Dutch Deep Red breeds, reflecting their common histories. A considerable amount of gene flow from Red Holstein to German Angler and to German Red and White Dual-Purpose was revealed, which is consistent with frequent crossbreeding to improve productivity of these local breeds. In Red Holstein, marked genomic signatures of selection were reported on chromosome 18, suggesting directed selection for important breeding goal traits. Furthermore, tests for signatures of selection between Red Holstein, Red and White Dual-Purpose, and Meuse-Rhine-Yssel uncovered signals for all investigated pairs of populations. The corresponding genomic regions, which were putatively under different selection pressures, harboured various genes which are associated with traits such as milk and beef production, mastitis and female fertility.

**Conclusions:**

This study provides comprehensive knowledge on the genetic constitution and genomic connectedness of divergent red cattle populations in Northern Europe. The results will help to design and optimize breeding strategies. A joint genomic evaluation including some of the breeds studied here seems feasible.

## Background

The genetic diversity of cattle breeds is shaped by evolutionary forces such as genetic drift, migration, selection and geographical separation. Livestock breeds differ greatly from natural populations since they have experienced strong human-mediated selection and directed mating decisions. As a result, a large number of highly specialized breeds has evolved to meet a variety of human needs [1]. Furthermore, all of these processes have left detectable traces in the genome of domestic livestock species [2]. The development of genome-wide marker (single nucleotide polymorphisms, SNPs) panels enables a reliable description of the genetic diversity and population structure in cattle breeds [3, 4]. In addition, the growing availability of genomic tools provides the opportunity to investigate gene flow and genetic connectedness among livestock populations on a molecular basis. Thus, valuable insights into historical breeding strategies are gained. At the same time, such information will help to improve the genetic management of current breeds [5].

In Northern Europe, various red cattle breeds with small to medium population sizes exist. While their occurrence is often confined to restricted geographical regions, such traditional breeds are known to be well adapted to their prevailing environmental conditions [6]. However, small-sized cattle populations are faced with some challenges. One of these challenges is the acceleration of genetic gain, which is crucial; otherwise, the performance gap to commercial cattle breeds (e.g., Holstein Friesian) will expand. As a consequence, local cattle breeds will become increasingly uncompetitive compared to conventional breeds [7] and, thus, not economically viable for farmers. In the past, traditional breeds have declined dramatically due to their replacement by highly productive breeds [8, 9]. Consequently, native populations commonly have a narrow genetic base, which limits opportunities for selection. Accordingly, a proper genetic management of numerically small breeds is particularly important to ensure their conservation [4].

The breeding programs of the Northern red dairy cattle breeds are primarily organized at the national level, and especially for the smaller populations, their aim is to maintain genetic diversity in order to keep the breeds viable. However, running breeding programs for small-sized populations managed by commercial breeding organizations is often expensive, inefficient, and returns of investment are limited. Thus, from an economic and practical point of view, cooperation across breeding companies that aim at the establishment of a common reference population for genomic prediction is expected to be advantageous [10]. Investments to create the required infrastructure for the implementation of genomic selection are shared by multiple breeding organizations, which makes initial costs manageable. Therefore, a collaboration across countries and breeds through a common genomic evaluation has the potential to ensure the conservation and a sustainable development in terms of genetic gain of these numerically smaller populations.

In large populations, genomic selection has accelerated genetic gain mainly by reducing the generation interval in dairy cattle breeding [11, 12]. However, accurately estimated genomic breeding values (GEBV) are crucial. For breeds that have a limited number of animals, implementation of genomic evaluation is not straightforward because the accuracy of GEBV depends largely on the size of the reference population [13, 14]. One strategy to overcome this problem is to set-up a multi-breed reference population composed of animals from several breeds [15–17]. Studies have shown that the utility of a multi-breed reference population is strongly affected by the genetic differentiation between the breeds included [18–20]. Therefore, prior information on genetic relationships is useful to assess the impact of the implementation of a multi-breed reference population.

Recently, various studies have successfully shed light on the patterns of genetic diversity and the relatedness of cattle populations in Europe and worldwide [21–24]. However, the knowledge on the genetic constitution of red cattle breeds from Northern Europe is still limited. The main objective of the current study was to characterize ten genetically divergent red cattle populations originating from Germany, The Netherlands and Denmark. These breeds are bounded in a common international project that aims at their promotion and ultimately their preservation. As a specific objective, a joint genomic evaluation is pursued. In this study, we used well-established population genetic analysis tools to assess the population structure and genetic diversity of these breeds.

## Methods

### Data and data processing

Genomic data of ten red cattle breeds from Germany (Red and White Dual-Purpose, German Angler, and Red Holstein), The Netherlands (Meuse-Rhine-Yssel, Deep Red, Dutch Red Friesian, Groningen White-Headed, Dutch Belted, and Improved Red) and Denmark (Traditional Danish Red) were available for the analyses in this study. Genomic data for German breeds were provided by the breeding organization Rinderzucht Schleswig–Holstein (RSH e.G.). The genotypes of Dutch cattle breeds were obtained from the Center for Genetic Resources (CGN) in The Netherlands, and genomic data of Traditional Danish Red were provided by Aarhus University. Animals had previously been genotyped with four SNP chips (Illumina BovineSNPv1, Illumina BovineSNPv2, Illumina BovineSNPv3 and Illumina BovineHD; Illumina Inc., San Diego, CA, USA). Detailed information on the samples is in Additional file 1: Table S1. To prepare the main dataset for our analyses, different data processing steps were conducted. First, data for all breeds were merged using the PLINK v1.09 software [25] by retaining SNPs common to the four arrays and excluding SNPs that were not assigned to autosomes. Physical positions of the SNPs were mapped according to the *Bos taurus* genome reference assembly ARS-UCD1.2 [26] available from [27]. Second, genotypes from this combined dataset were quality-controlled based on the following criteria: (1) animals with a SNP call rate lower than 90% were removed (–mind 0.1) and (2) SNPs with more than 10% missing data were discarded (–geno 0.1). Filtration for minor allele frequency was not applied at this stage. Thus, the resulting combined dataset included 1425 genotypes and 36,195 SNPs spanning the 29 bovine autosomes. The average distance between consecutive SNPs was 69.0 kb. For some of the subsequent analyses, this dataset was further processed as described in the following sections. Details on the final datasets are summarized in Additional file 2: Table S2.

### Genetic diversity indices and runs of homozygosity

In order to assess within-breed genetic variation, the following parameters i.e., average minor allele frequency (MAF), average observed heterozygosity (H_o_) and average expected heterozygosity (H_e_), were estimated with PLINK v1.09 [25] using the main dataset that contained 1425 individuals and 36,195 SNPs. To quantify the genomic autozygosity at the population level, runs of homozygosity (ROH) were detected using the same dataset split according to breed. Furthermore, individual autozygosity (${F}_{ROH}$) was computed as the ratio of the length of all ROH (${L}_{ROH}$) beyond a specific threshold (> 4 Mb) and the total length of the autosomal genome (${L}_{AUTO}$) covered by SNPs following McQuillan et al. [28]:

$${F}_{ROH}= \frac{\sum {L}_{ROH}}{{L}_{AUTO}}.$$

We set the threshold for the length of ROH at 4 Mb because the number of shorter homozygous segments is systematically overestimated when using a 50 K SNP array [29]. The total length of the autosomal genome covered by SNPs was 2,498,774 kb. ROH were detected using the cgaTOH Clustering Suite v1.0 [30] with a sliding window approach and the following parameters set for the identification and characterization of ROH: (1) a minimum physical length of 4000 kb for a ROH, (2) a minimal number of consecutive homozygous SNPs of 40, and (3) a maximum physical gap of 1000 kb between consecutive homozygous SNPs. For ROH shorter than 16 Mb, no heterozygous SNPs were allowed, whereas for ROH longer than 16 Mb one heterozygous SNP was permitted. As suggested by Ferenčaković et al. [31], the maximum number of missing SNPs was set as a function of the length of ROH with 1, 2 and 4 missing genotypes allowed for ROH in class sizes > 4 Mb, > 8 Mb and > 16 Mb, respectively. For the calculation of ${F}_{ROH}$, all detected ROH longer than 4 Mb were considered.

### Estimation of genome-wide linkage disequilibrium

To explore the overall levels of linkage disequilibrium (LD) in the breeds studied, the genome-wide pairwise LD was estimated for SNPs on the same chromosome and less than 2 Mb apart using PLINK v1.09 [25]. LD was measured as the squared correlation (*r*^*2*^) between the alleles at two loci according to Hill and Robertson [32]. For the graphical representation, SNP distances were collected in bins of 100 kb and the average *r*^*2*^ in each bin was plotted.

### Population structure and admixture

To investigate the population structure and genomic variability within and across cattle breeds and to detect admixture, we applied three complementary approaches: principal component analysis (PCA), ADMIXTURE and TreeMix. In order to avoid oversampling of some breeds for the analyses of population structure [33], a dataset with at most 50 randomly selected individuals per breed was created. For PCA, the final dataset consisted of 394 individuals from ten cattle breeds and 36,195 SNPs. PCA was performed using the smartPCA component of the EIGENSOFT 5.0 software [34, 35]. To visualize the results, PCA plots were created using R version 3.6.3 (R Development Core Team, 2020) and ggplot2 [36]. In addition, we investigated population structure by using the unsupervised model-based clustering approach implemented in ADMIXTURE version 1.23 [37]. In order to limit pairwise LD and overrepresentation of genomic regions with high SNP density on the chips used, the dataset was arbitrary thinned using the—bp-space option in PLINK v1.09 [25]. The threshold for thinning was calculated as the total length of the autosomal genome divided by the number of SNPs (2,498,774 bp/36,195 = 69 bp). Consequently, one SNP from each pair of SNPs closer than the given threshold was randomly removed. After thinning, 19,717 SNPs remained. A preliminary run of ADMIXTURE ensured the identification of possibly misclassified animals. Consequently, one animal of the Dutch Belted breed was removed due to a high proportion of admixed ancestry, while the remaining animals hardly showed any admixture. For the final analysis, a dataset including 393 individuals was used. The most likely number of ancestral populations (K) for the given dataset was determined using cross-validation. Therefore, a 10-fold cross-validation for K values ranging from 2 to 40 was conducted. The ADMIXTURE results were visualized using POPHELPER [38].

A maximum likelihood based phylogenetic tree was constructed using TreeMix v1.13 [39] to evaluate population splits and gene flow among the cattle breeds. In order to infer statements on the relationships among the breeds from Germany, The Netherlands and Denmark and other European cattle breeds, for the TreeMix analysis, we enlarged this dataset with genotypes of 12 reference breeds, which are publicly available from the WIDDE database (Web-Interfaced next generation database for genetic diversity exploration [40]) and previously reported in [41–44]. Detailed information on these breeds is in Additional file 1: Table S1. The resulting dataset consisted of 678 individuals from 22 breeds and 35,101 SNPs after quality control. As for the ADMIXTURE analysis, pairwise LD was limited by data thinning. The threshold was computed as follows: 2,498,774 bp/35,101 = 71 bp. After thinning, 19,294 SNPs remained (see Additional file 2: Table S2). As outgroup for the TreeMix analysis, which roots the tree and is usually a population that diverges largely from the remaining populations [45], we used the West African N’Dama (an African *Bos taurus* breed). Phylogenetic trees with migration events from 0 to 10 were constructed. The number of migration events that best fitted the data was identified using the fraction of the variance in the sample covariance matrix explained by the model covariance matrix [39]. Each analysis was run with 1000 bootstrap replicates in order to verify the consistency of the trees’ edges and nodes. In addition, to assess the robustness of the tree, the analysis was repeated three times for each number of allowed migration events. Visualization of the TreeMix results was performed using the BITE R package [46]. Furthermore, to test for admixture among breeds and to assess the statistical significance of migration events, the THREEPOP function (implemented in TreeMix) was run to calculate the f3-statistics [47]. This allowed us to test whether one target population descended from two source populations. A strongly negative *z* score indicates that a population has arisen by admixture of the two source populations. For the calculation of the f3-statistics, for all possible combinations of populations, blocks of 1000 SNPs were used and a Bonferroni adjustment of the p-value was applied.

To gain additional insight into the genetic relationships among the populations and to measure the degree of population differentiation, average genome-wide F_ST_ values were estimated using the estimator of Weir and Cockerham [48] in PLINK v1.09 [25]. Genetic differentiation was investigated between pairs of all available breeds (including breeds from the WIDDE database). For this purpose, the merged dataset from the TreeMix analysis prior to thinning was used, which comprised 678 individuals and 35,101 SNPs (Additional file 2: Table S2).

### Haplotype-based analyses: detection of selection signatures

Recurrent artificial selection can result in signatures of selection in the genome [49], which can be detected because they are anticipated to deviate from the expectation under the neutral theory [50]. The neutral theory states that most of the genetic variants have no impact on an individual’s performance. Under recurrent selection pressure, the frequency of a favored allele can rapidly increase in a population. As selection acts, over time the genomic region in LD with the allele under directional selection becomes marked by reduced allelic diversity. Thus, signatures of selection are characterized by typical patterns of DNA, namely by particular alleles that are surrounded by frequent, long-range haplotypes [51]. In our study, we used the haplotype-based integrated haplotype score (iHS) test [52] to detect putative signatures of selection in the Red and White Dual-Purpose, Meuse-Rhine-Yssel, and Red Holstein breeds. To compare these breeds, we applied the cross population extended haplotype homozygosity (XPEHH) test [53] between all combinations of these three populations. For both analyses, we used the combined and quality-filtered dataset that included 1124 individuals of the three populations and 36,195 SNPs. The genotype data of each breed was split by chromosome and phased using SHAPEIT2 [54]. To correct for local differences in recombination rate, a genetic marker map of the cattle genome [55] was used for phasing. Both $\mathrm{iHS}$ and $\mathrm{XPEHH}$ were calculated for each autosomal SNP using the R package *rehh* [56]. Information on ancestral and derived alleles for the investigated SNPs to compute $\mathrm{iHS}$ was obtained from Rocha et al. [57]. According to Voight et al. [52], the standardized $\mathrm{iHS}$ values were calculated as follows:

$$\mathrm{iHS}=\frac{ln\left(\frac{{iHH}_{1}}{{iHH}_{0}}\right)- E\left[ln\left(\frac{{iHH}_{1}}{{iHH}_{0}}\right)\right]}{SD\left[ln\left(\frac{{iHH}_{1}}{{iHH}_{0}}\right)\right]},$$

where ${iHH}_{1}$ and ${iHH}_{0}$ are the integrated haplotype homozygosity for the ancestral and derived allele, respectively, and $E\left[ln\left(\frac{{iHH}_{1}}{{iHH}_{0}}\right)\right]$ is the average of $ln\left(\frac{{iHH}_{1}}{{iHH}_{0}}\right)$ with its standard deviation $SD\left[ln\left(\frac{{iHH}_{1}}{{iHH}_{0}}\right)\right]$. Subsequently, the genome was divided in non-overlapping windows of 500 kb, and for each segment, the average $\left|\mathrm{iHS}\right|$ score was calculated as previously done by Qanbari et al. [2]. Candidate regions of positive selection were defined as the top 0.5% windows with the highest $\left|\mathrm{iHS}\right|$ scores.

The cross-population $\mathrm{XPEHH}$ statistic, which was introduced by Sabeti et al. [53], was used to detect differences in signatures of selection between populations. To calculate $\mathrm{XPEHH}$, the integrated haplotype homozygosity for population $A$ (${iHH}_{A}$) and population $B$ (${iHH}_{B}$) was calculated at each SNP. Then, the standardized $\mathrm{XPEHH}$ values were calculated as follows:

$$\mathrm{XPEHH}=\frac{ln\left(\frac{{iHH}_{A}}{{iHH}_{B}}\right)- E\left[ln\left(\frac{{iHH}_{A}}{{iHH}_{B}}\right)\right]}{SD\left[ln\left(\frac{{iHH}_{A}}{{iHH}_{B}}\right)\right]},$$

where $E\left[ln\left(\frac{{iHH}_{A}}{{iHH}_{B}}\right)\right]$ is the average of $ln\left(\frac{{iHH}_{A}}{{iHH}_{B}}\right)$ with its standard deviation $SD\left[ln\left(\frac{{iHH}_{A}}{{iHH}_{B}}\right)\right]$.

As for $\mathrm{iHS}$, the average $\left|\mathrm{XPEHH}\right|$ values of all SNPs in non-overlapping windows of 500 kb were computed and the 0.5% of segments with the highest $\left|\mathrm{XPEHH}\right|$ scores were identified as putative signatures of selection.

To visualize the detected signatures of selection, average $\left|\mathrm{iHS}\right|$ and $\left|\mathrm{XPEHH}\right|$ values per window were plotted against their physical position on each chromosome. Annotated genes within the genomic regions putatively under selection were identified using the NCBI Genome Data Viewer [58].

## Results

### Genetic diversity and runs of homozygosity

The estimated genetic diversity indices for the ten red breeds are in Table 1. Average MAF was lowest for the Traditional Danish Red (0.222 ± 0.158) and highest for the German Angler breed (0.281 ± 0.131). Average H_o_ ranged from 0.303 ± 0.189 (Traditional Danish Red) to 0.375 ± 0.129 (German Angler). Similarly, H_e_ was lowest for the Traditional Danish Red (0.296 ± 0.176) and Groningen White-Headed (0.302 ± 0.172) breeds, and highest for the German Angler (0.369 ± 0.122) and Red Holstein (0.359 ± 0.132) breeds indicating that Traditional Danish Red and Groningen White-Headed are less diverse populations.

**Table 1:** Genetic diversity indices for the red cattle breeds under study

Breed	Average MAF ± SD	Average H_o_ ± SD	Average H_e_ ± SD
German Angler	0.281 ± 0.131	0.375 ± 0.129	0.369 ± 0.122
Dutch Belted	0.245 ± 0.152	0.335 ± 0.191	0.323 ± 0.163
Dutch Friesian Red	0.255 ± 0.146	0.342 ± 0.163	0.337 ± 0.152
Deep Red	0.260 ± 0.145	0.349 ± 0.173	0.343 ± 0.149
Groningen White Headed	0.227 ± 0.156	0.313 ± 0.189	0.302 ± 0.172
Improved Red	0.270 ± 0.141	0.369 ± 0.170	0.354 ± 0.142
Meuse-Rhine-Yssel	0.257 ± 0.145	0.344 ± 0.153	0.339 ± 0.149
Traditional Danish Red	0.222 ± 0.158	0.303 ± 0.189	0.296 ± 0.176
Red and White Dual-Purpose	0.266 ± 0.139	0.356 ± 0.142	0.351 ± 0.139
Red Holstein	0.272 ± 0.136	0.363 ± 0.139	0.359 ± 0.132

For each breed, the mean and standard deviation (SD) of minor allele frequency (MAF), observed heterozygosity (H_o_) and expected heterozygosity (H_e_) is presented

**Fig. 1:**
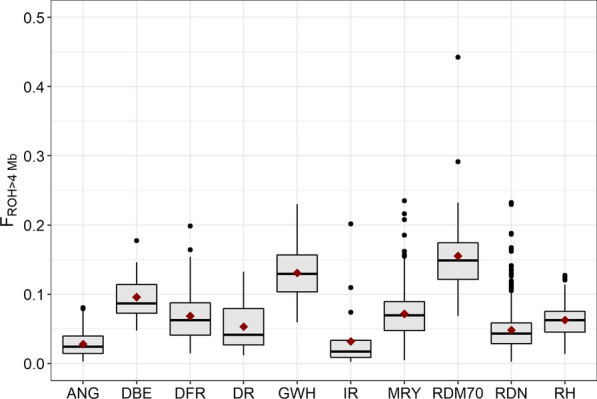
Boxplots showing the level of genomic inbreeding (F_ROH>4 Mb_) per breed. Red dots indicate the mean values. *ANG* German Angler, *DBE* Dutch Belted, *DFR* Dutch Friesian Red, *DR* Deep Red, *GWH* Groningen White-Headed, *IR* Improved Red, *MRY* Meuse-Rhine-Yssel, *RDM70* Traditional Danish Red, *RDN* Red and White Dual-Purpose, *RH* Red Holstein

ROH were detected in all breeds, but their length and frequency differed among populations. Additional file 3: Table S3 presents the basic statistics: average number of ROH per breed and average length of ROH per breed for different size classes. The average number of ROH (> 4 Mb) per breed was largest for the Traditional Danish Red (40.8) and Groningen White-Headed (35.3) breeds, which also showed the largest mean number of ROH per breed in all other length categories. In contrast, the Improved Red (7.3) and German Angler (7.7) breeds exhibited the smallest mean number of ROH (> 4 Mb) per breed. For the other breeds, the average number of ROH (> 4 Mb) ranged from 12.3 to 23.1. Likewise, the average total length of ROH (> 4 Mb) per breed was largest for the Traditional Danish Red (388.3 Mb) and Groningen White-Headed (327.1 Mb) breeds, and smallest for the German Angler (69.9 Mb) and Improved Red (79.8 Mb). For the other cattle breeds, the mean length of ROH (> 4 Mb) per breed ranged from 120.6 to 239.5 Mb. For the long ROH category (> 16 Mb), the mean length was longest in Improved Red (138.3 Mb ± 148.2), resulting from the small sample size, which included two individuals with extremely long ROH. Comparison of the Red and White Dual-Purpose and Meuse-Rhine-Yssel breeds showed that the latter has both a larger average number of ROH and markedly greater length of ROH in all size categories. The level of genomic inbreeding (${F}_{ROH}$) based on ROH length > 4 Mb varied within and among breeds (Fig. 1; Additional file 4: Table S4). The highest average inbreeding coefficients were observed for Traditional Danish Red, with the genomic inbreeding coefficient ranging from 0.052 for ${F}_{ROH>16\mathrm{Mb}}$ to 0.155 for ${F}_{ROH>4\mathrm{Mb}}$. In addition, the two most inbred animals belonged to this breed and had 44.2% (1105.23 Mb) and 29.1% (727.63 Mb) of their genome included in ROH. The lowest level of genomic inbreeding was observed for German Angler, with mean inbreeding coefficients ranging from 0.017 for ${F}_{ROH>16\mathrm{Mb}}$ to 0.028 for ${F}_{ROH>4\mathrm{Mb}}$.

### Estimation of genome-wide linkage disequilibrium

The LD decay as a function of inter-marker distance differed between breeds (Additional file 5: Figure S1). Decay of LD was slowest for the Dutch Belted, Groningen White-Headed, and Traditional Danish Red breeds. Their decay curves clearly differed from those of the other breeds, suggesting that levels of inbreeding are higher for the breeds Dutch Belted, Groningen White-Headed, and Traditional Danish Red. For markers in close physical proximity (< 300 kb), Meuse-Rhine-Yssel and Red and White Dual-Purpose showed similar average LD, but for those separated by more than 300 kb, the average LD was slightly lower in Red and White Dual-Purpose than in Meuse-Rhine-Yssel. In the German Angler breed, the gradual decrease in LD was fastest and the long-range LD was lowest, indicating high degrees of admixture.

### Population structure

**Fig. 2:**
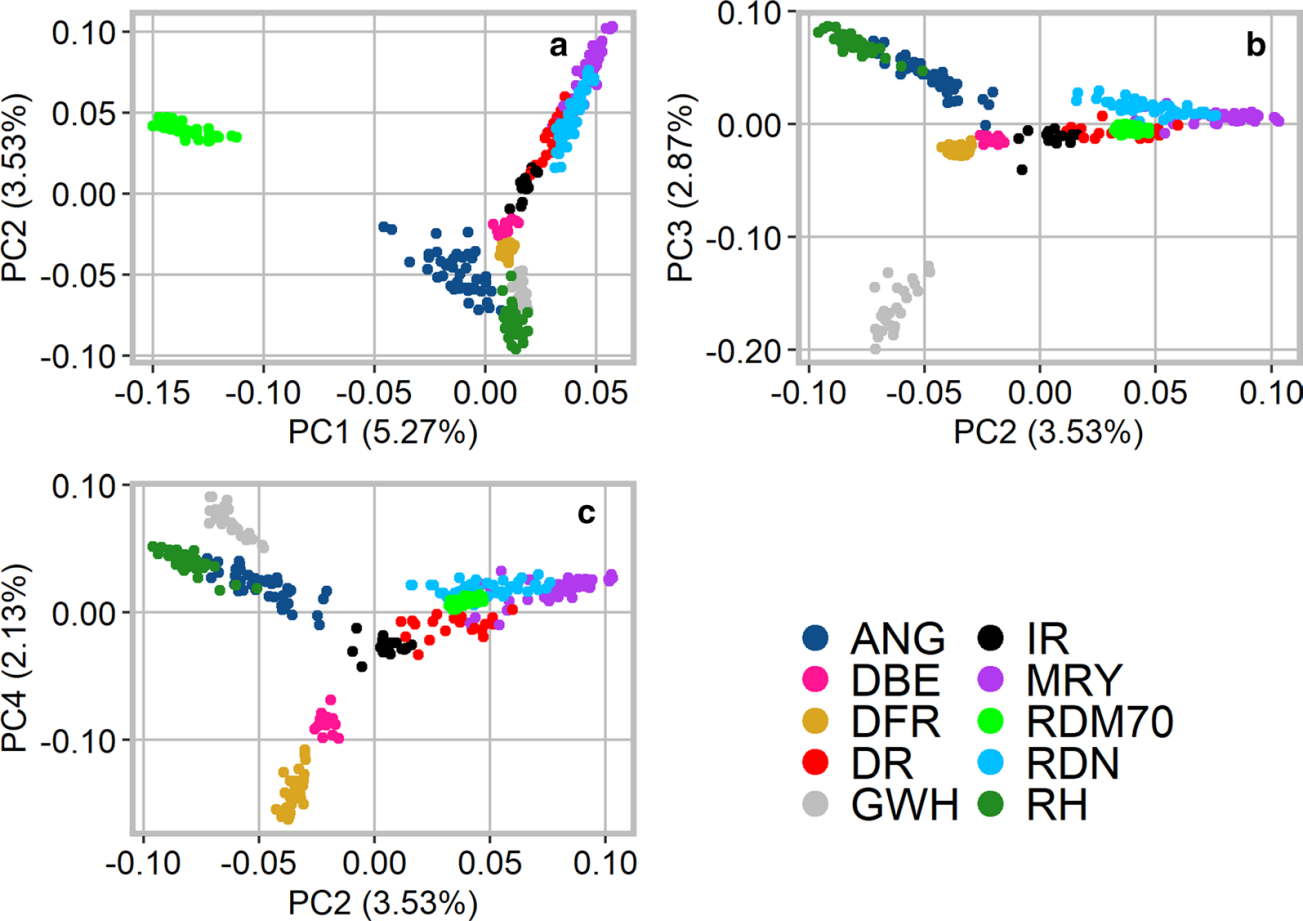
Genetic relatedness among the cattle breeds from Germany, The Netherlands and Denmark using principal component analysis (**a** PC1 vs. PC2; **b** PC2 vs. PC3; **c** PC2 vs. PC4). *ANG* German Angler, *DBE* Dutch Belted, *DFR* Dutch Friesian Red, *DR* Deep Red, *GWH* Groningen White-Headed, *IR* Improved Red, *MRY* Meuse-Rhine-Yssel, *RDM70* Traditional Danish Red, *RDN* Red and White Dual-Purpose, *RH* Red Holstein

To assess relationships among the breeds and their genetic diversity, PCA was performed. The ten first principal components (PC) explained 18.9% of the total variance in the given dataset. PC1 explained 5.27% of the total variance and clearly separated Traditional Danish Red from the other breeds (Fig. 2). Moreover, German Angler showed a deviation from zero on PC1. PC2 explained 3.53% of the variance and separated the Dutch Meuse-Rhine-Yssel, Deep Red, Improved Red breeds, and the German Red and White Dual-Purpose from the remaining breeds, i.e. Dutch Belted, Dutch Friesian Red, Red Holstein, Groningen White-Headed, and German Angler. In addition, the results showed a large overlap between Meuse-Rhine-Yssel, Red and White Dual-Purpose and, in part, Deep Red. By plotting PC2 against PC3, which explained 2.87% of the variance, we observed the separation of the Groningen White-Headed breed. Furthermore, both the German Angler and Red Holstein breeds separated on PC3 and formed a distinct cluster. On PC4, which explained 2.13% of the total variance, Dutch Belted and Dutch Friesian Red were clearly differentiated. The results of the PCA including 11 additional breeds from the WIDDE database are illustrated on Additional file 6: Figure S2, and are not discussed in this paper.

**Fig. 3:**
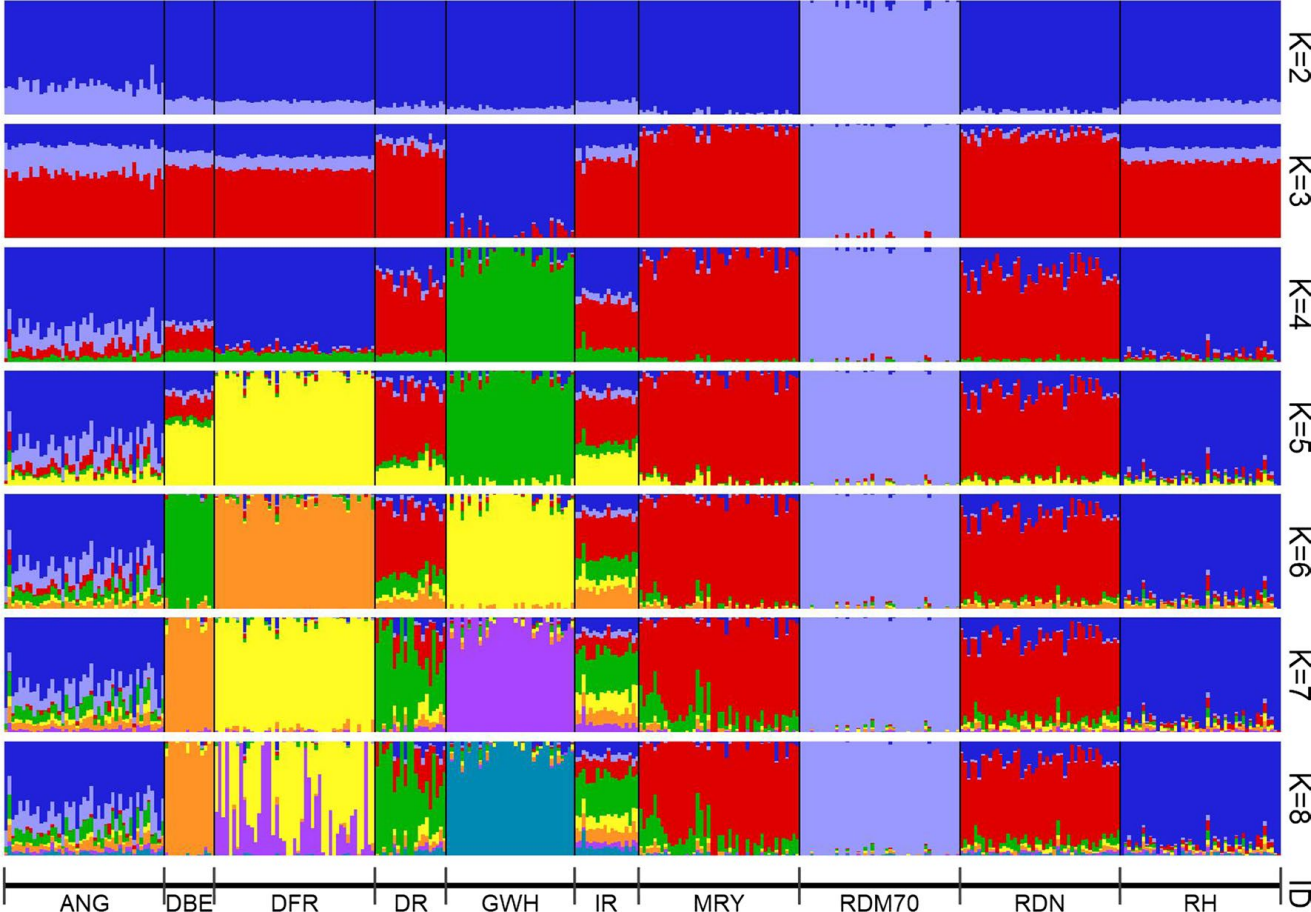
Unsupervised model-based clustering results of 393 individuals using 19,717 SNPs. Presented are K values from 2 to 8. *ANG* German Angler, *DBE* Dutch Belted, *DFR* Dutch Friesian Red, *DR* Deep Red, *GWH* Groningen White-Headed, *IR* Improved Red, *MRY* Meuse-Rhine-Yssel, *RDM70* Traditional Danish Red, *RDN* Red and White Dual-Purpose, *RH* Red Holstein

The ADMIXTURE analysis provided insights into the genetic ancestry of the cattle breeds studied. The implemented ten-fold cross-validation analysis revealed an optimal number of eight ancestral populations for the given dataset (see Additional file 7: Figure S3). The ADMIXTURE graphs for K = 2 to 8 are in Fig. 3. For all values of K, a clear genetic differentiation was found for the Traditional Danish Red breed that also showed the lowest level of admixture. At K = 3, Groningen White-Headed was separated with a distinct ancestral component, and at K = 5, the Dutch Friesian Red and Red Holstein breeds were separated each with their own genetic ancestries. At the optimal number of ancestral populations (K = 8), the Dutch Belted, Dutch Friesian Red, Deep Red, Groningen White-Headed, Traditional Danish Red, and Red Holstein breeds were each characterized by specific ancestry components. The Meuse-Rhine-Yssel and Red and White Dual-Purpose breeds displayed similar patterns of ancestry. However, the German dual-purpose breed comprised a larger contribution from the Red Holstein component than Meuse-Rhine-Yssel. Whereas all Red and White Dual-Purpose individuals revealed shared ancestry with Red Holstein, only a few Meuse-Rhine-Yssel animals showed the Red Holstein component in the ADMIXTURE analysis. The German Angler breed did not form an ancestry component of its own. In fact, German Angler was characterized by a high degree of admixture and shared a large part of its ancestry with Red Holstein. In addition, most German Angler animals showed considerable proportions of the Traditional Danish Red component. The Improved Red breed was characterized by high levels of admixture in all animals. However, even at increased K-values, Improved Red was not assigned a separate ancestral component (see Additional file 8: Figure S4), which implies either that the ADMIXTURE algorithm was not able to calculate the genetic ancestry of Improved Red in a reasonable manner, or that this breed is, in fact, highly admixed. The results of the ADMIXTURE analysis including the 11 additional breeds from the WIDDE database are provided in Additional file 9: Figure S5.

**Fig. 4:**
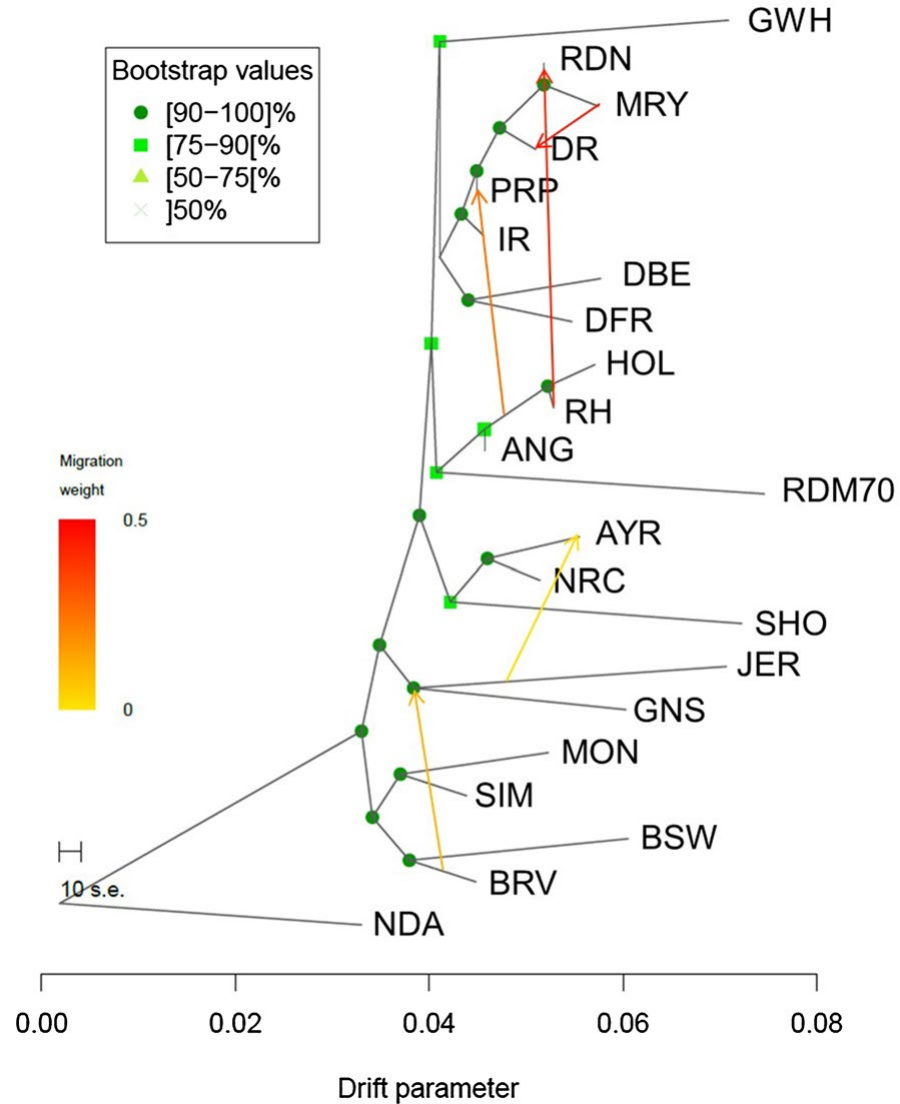
Phylogenetic tree of 22 cattle breeds with five migration events constructed using the TreeMix software. *ANG* German Angler, *AYR* Finnish Ayrshire, *BRV* Braunvieh, *BSW* Brown Swiss, *DBE* Dutch Belted, *DFR* Dutch Friesian Red, *DR* Deep Red, *GNS* Guernsey, *GWH* Groningen White-Headed, *HOL* Holstein Friesian, *IR* Improved Red, *JER* Jersey, *MON* Montbéliarde, *MRY* Meuse-Rhine-Yssel, *NDA* N’Dama, *NRC* Norwegian Red Cattle, *PRP* French Red Pied Lowland, *RDM70* Traditional Danish Red, *RDN* Red and White Dual-Purpose, *RH* Red Holstein, *SHO* Shorthorn, *SIM* Simmental Cattle

A phylogenetic tree was constructed to infer population splits and gene flow among 21 European cattle breeds. Without any migration events, the model explained 90.3% of the variance (see Additional file 10: Figure S6). All the models that were run by allowing migrations explained a higher proportion of the variance. The f-index suggested that the tree with five migration events was the most suitable with the amount of explained variance reaching 97.5%. A further increase in the number of migration events improved the model fit only marginally. The results of the model with five migration events are shown in Fig. 4. The bootstrap values of most edges ranged from 90 to 100% and indicate the proportion of 1000 bootstrap replicates that reproduced each branch point. The phylogenetic tree divided the cattle breeds into five groups. The first group consisted of all the Dutch breeds under study, the German Red and White Dual-Purpose, and the French Red Pied Lowland breeds. Within this group, further differentiation was observed with two separate clades i.e. one including Meuse-Rhine-Yssel, Red and White Dual-Purpose, Deep Red, French Red Pied Lowland and Improved Red, and one including the Dutch Belted and Dutch Friesian Red breeds. The Groningen White-Headed formed a separate clade and showed the largest amount of genetic drift among these eight breeds. The second group consisted of the Holstein Friesian, Red Holstein and German Angler breeds, which formed one clade, with the Traditional Danish Red being as sister to this clade and having the largest drift parameter among all the breeds included in the TreeMix analysis. The third group was formed by the Norwegian Red Cattle, Finnish Ayrshire and Shorthorn breeds, the fourth group consisted of the Channel Island breeds Guernsey and Jersey, and the fifth group included the breeds that had a geographical origin in the Alpine area (Montbeliarde, Simmental, Braunvieh, and Brown Swiss) with a close proximity between Simmental and Montbeliarde, and between Braunvieh and Brown Swiss. The five migration events identified by TreeMix had different migration weights. The strongest migration was found to occur from the Holstein-influenced group to German Red and White Dual-Purpose, and from Meuse-Rhine-Yssel to Deep Red. The third migration event indicated gene flow from the Holstein group to French Red Pied Lowland. In addition, the results showed migration events from Braunvieh to the Channel Island breeds and from Jersey to Finnish Ayrshire. The f3-statistics were significantly negative for the German Angler, Red and White Dual-Purpose, and French Red Pied Lowland breeds as target populations and for a total of 43 migration events [Bonferroni-corrected threshold of 0.0026 was obtained by 0.05/(19,294 SNPs/1000 SNPs per block)]. The f3-statistics with the highest negative *z* scores revealed gene flow from Red Holstein and Traditional Danish Red to German Angler (*z* score = − 18.42) and from Red Holstein and Meuse-Rhine-Yssel to Red and White Dual-Purpose (*z* score = − 15.30). In addition, a migration event from either Holstein Friesian or Meuse-Rhine-Yssel to French Red Pied Lowland (*z* score = − 10.77) was confirmed by the f3-statistics (see Additional file 11: Table S5).

### F_ST_ values

We estimated the genome-wide F_ST_ values [48] to measure the degree of genetic differentiation among the populations studied. F_ST_ values ranged from 0.014 (between Red and White Dual-Purpose and Meuse-Rhine-Yssel) to 0.168 (between Traditional Danish Red and Improved Red) (see Additional file 12: Table S6). In general, the degree of genetic differentiation was highest for the Jersey, Traditional Danish Red, and Shorthorn breeds compared to all the other breeds. In contrast, F_ST_ values were low between French Red Pied Lowland and Improved Red (0.017), French Red Pied Lowland and Deep Red (0.018), Red and White Dual-Purpose and French Red Pied Lowland (0.018), and Holstein Friesian and Red Holstein (0.018). In addition, the genetic differentiation between Red Holstein and German Angler was low (F_ST_ = 0.021) and that between Red Holstein and Red and White Dual-Purpose (F_ST_ = 0.053) was slightly lower than that between Red Holstein and Meuse-Rhine-Yssel (F_ST_ = 0.082).

### Detection of signatures of selection

**Fig. 5:**
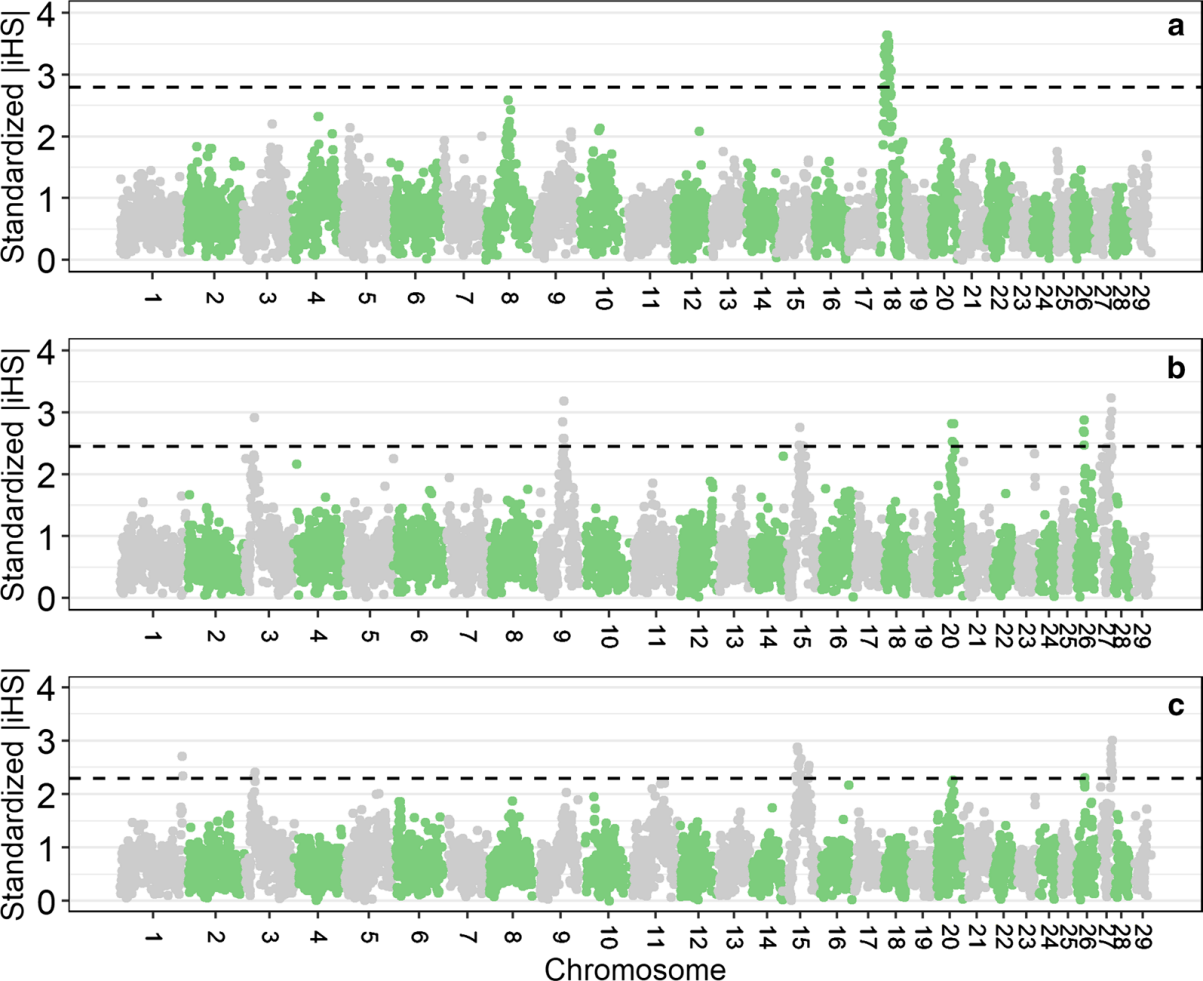
Genome-wide distribution of standardized |iHS| values averaged in windows of 500 kb per chromosome for the populations Red Holstein (**a**), Red and White Dual-Purpose (**b**) and Meuse-Rhine-Yssel (**c**). Dashed lines indicate the cut-off values representing 0.5% of windows with highest standardized |iHS|

For the Red Holstein, Red and White Dual-Purpose, and Meuse-Rhine-Yssel breeds, putative signatures of selection are in Table 2; this table also indicates some of the annotated genes that are present within the identified genomic regions [for a full list of annotated genes (see Additional file 13: Table S7)]. In order to visualize the results, Fig. 5 shows the genome-wide standardized $\left|\mathrm{iHS}\right|$ scores averaged across 500 kb-windows. In Red Holstein, a set of recognizable signatures of selection was found on *Bos taurus* (BTA) chromosome 18 between 16.5 and 33.5 Mb. The annotated genes on BTA18 include *ZNF423* (16.5–18.0 Mb), *ADCY7*, *NKDI*, *BRD7* (18.5–19.0 Mb), *FTO*, *IRX3*, *IRX6*, and *RPGRIP1L* (21.5–26.0 Mb). In the German dual-purpose breed, the strongest evidence of selection was found on six chromosomes: BTA3, 9, 15, 20, 26 and 27. On BTA9, several genes are mapped within the identified genomic region (59.0–62.5 Mb), e.g., *BACH2*, *RARS2*, *SLC35A1*, and *CFAP206*. In the Red and White Dual-Purpose breed, putative signals of positive selection were detected on BTA15, which contained the *KCNJ11* and *NUCB2* genes (35.0–37.0 Mb) and the *EIF4G2* and *SBF2* genes (41.5–42.5 Mb). In addition, the results revealed variants in the genomic region from 39.0 to 41.0 Mb on BTA20, which is assumed to be under positive selection pressure and which includes the *RAI14*, *SLC45A2*, *TTC23L* and *ADAMTS12* genes. A genomic region (21.5–23.5 Mb) under putative positive selection on BTA26 harbors the *SLF2*, *LBX1* and *BTRC* genes. A signature of selection on BTA27 (30.5–34.5 Mb) contains the genes *UNC5D* and *KCNUI*. In the Dutch Meuse-Rhine-Yssel, the strongest signatures of selection were located on three chromosomes: BTA1 (148.5–150.0 Mb), a region that carries the *PIPG* gene, BTA3 (22.0–26.5 Mb), and BTA15 with the 34.0–37.0 Mb region containing the *GRAMD1B* gene and the 9.0–42.5 Mb region containing the *EIF4G2* and *ZBED5* genes.

**Table 2:** Genomic regions associated with top 0.5% of integrated haplotype score (|iHS|) values and annotated genes for Red Holstein (RH), Red and White Dual-Purpose (RDN) and Meuse-Rhine-Yssel (MRY)

Breed	BTA	Region (Mb)	Average |iHS|	Annotated genes
RH	18	16.5–18.0	3.05	*ZNF423*
	18	18.5–19.0	3.46	*ADCY7, NKD1, BRD7*
	18	20.0–20.5	3.00	
	18	21.5–26.0	3.32	*FTO, IRX3, IRX6, RPGRIP1L*
	18	26.5–30.0	3.20	
	18	33.0–33.5	3.07	
RDN	3	25.5–26.0	2.92	
	9	59.0–62.5	2.78	*BACH2, RARS2, SLC35A1, CFAP206*
	15	35.0–37.0	2.62	*KCNJ11, NUCB2*
	15	41.5–42.5	2.47	*EIF4G2, SBF2*
	15	44.5–45.0	2.46	
	20	39.0–41.0	2.68	*RAI14, SLC45A2, TTC23L, ADAMTS12*
	20	43.5–45.0	2.67	
	26	21.5–23.5	2.68	*SLF2, LBX1, BTRC*
	27	30.5–34.5	2.94	*UNC5D, KCNU1*
MRY	1	148.5–150.0	2.53	*PIGP*
	3	22.0–22.5	2.32	
	3	25.5–26.5	2.41	
	15	29.5–30.0	2.34	
	15	34.0–37.0	2.71	*GRAMD1B*
	15	39.0–42.5	2.55	*EIF4G2, ZBED5*
	15	59.0–60.5	2.50	

iHS| values were averaged in non-overlapping windows of 500 kb and adjacent windows were pooled

**Fig. 6:**
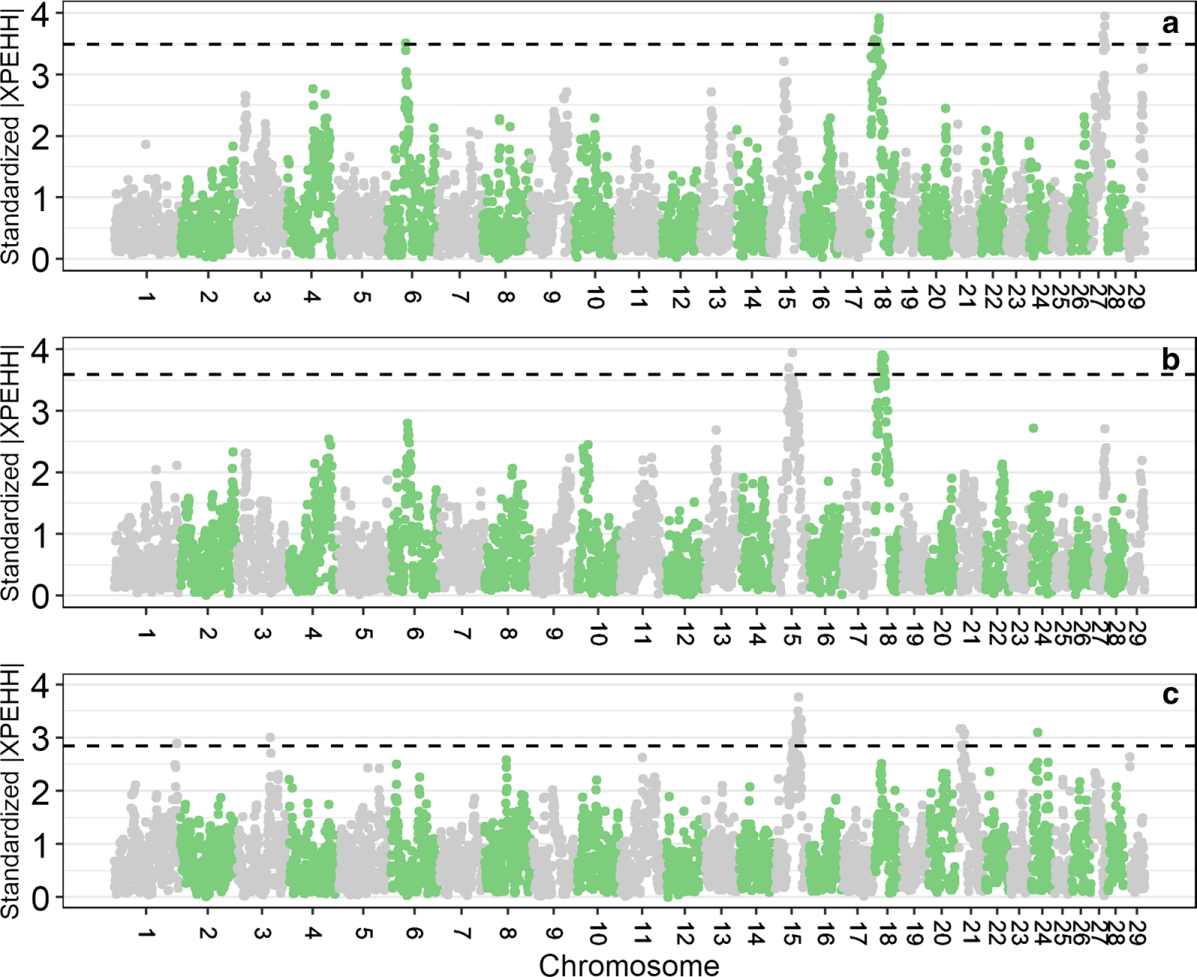
Genome-wide distribution of standardized |XPEHH| values averaged in windows of 500 kb per chromosome for the comparison of Red Holstein and Red and White Dual-Purpose (**a**), Red Holstein and Meuse-Rhine-Yssel (**b**) and Red and White Dual-Purpose and Meuse-Rhine-Yssel (**c**). Dashed lines indicate the cut-off values representing windows with 0.5% highest |XPEHH| scores

As Fig. 6 shows, the XPEHH-analyses revealed signatures of selection, i.e. differences in fixed alleles, for all investigated pairs of populations. Detailed information on physical positions of the detected signatures of selection that show the most extreme $|\mathrm{XPEHH}|$ scores along with the annotated genes in nearby genomic regions is in Table 3 [for a full list of annotated genes (see Additional file 13: Table S7)]. The analysis between Red Holstein and Red and White Dual-Purpose uncovered signals on BTA6, 18 and 27. On BTA18, the signal between 14.0 and 15.5 Mb comprised the *MC1R* gene. Furthermore, the region between 16.0 and 23.0 Mb contains several genes, e.g., *ZNF423*, *ADCY7*, *NKD1*, *BRD7*, *FTO*, *IRX3* and *RPGRIP1L*. By comparing the Red Holstein and Meuse-Rhine-Yssel populations, we detected signatures of selection on BTA15 and 18. On BTA18, the signatures of selection between 15.0 and 15.5 Mb included the *VPS35* gene and between 25.0 and 27.0 Mb the *NDRG4* gene. Interestingly, the comparisons of Red Holstein with either Red and White Dual-Purpose or Meuse-Rhine-Yssel, showed that they shared some signatures of selection on BTA18 between 16.0 and 24.0 Mb. However, the $|\mathrm{XPEHH}|$ scores were higher in the comparison between Red Holstein and Meuse-Rhine-Yssel than between Red Holstein and Red and White Dual-Purpose. XPEHH-analysis of the Red and White Dual-Purpose and Meuse-Rhine-Yssel populations detected signatures of selection on BTA1, 3, 15, 21 and 24. On BTA15, candidate genes are *ZNF215* in the 45.5–46.0 Mb region and *P4HA3*, *KCNE3*, *CHRDL2* and *NEU3* in the 53.5–56.5 Mb region, and on BTA21, two signatures of selection were found, i.e. one between 12.5 and 13.0 Mb with the candidate genes *MCTP2* and one between 21.5 and 22.0 Mb with the candidate gene *IQGAP1*.

**Table 3:** Genomic regions associated with top 0.5% of cross-population extended haplotype homozygosity (|XPEHH|) scores and annotated genes for the comparison of Red Holstein and Red and White Dual-Purpose (RH and RDN), Red Holstein and Meuse-Rhine-Yssel (RH and MRY) and Red and White Dual-Purpose and Meuse-Rhine-Yssel (RDN and MRY)

XPEHH-analysis between	BTA	Region (Mb)	Average |XPEHH|	Annotated genes
RH and RDN	6	45.0–45.5	3.51	
	18	14.0–15.5	3.87	*MC1R*
	18	16.0–19.0	4.28	*ZNF423, ADCY7, NKD1, BRD7*
	18	21.5–23.0	3.52	*FTO, IRX3, RPGRIP1L*
	18	25.0–27.5	3.87	
	27	32.5–33.0	3.65	*ZNF703*
	27	35.0–35.5	3.55	
	27	36.0–37.5	3.88	*GOLGA7, GPAT4*
RH and MRY	15	36.0–40.0	3.71	
	15	44.5–45.5	3.96	
	18	15.0–15.5	4.10	*VPS35*
	18	16.0–19.0	4.74	*ZNF423, ADCY7, NKD1, BRD7*
	18	21.0–24.0	3.82	*FTO, IRX3, RPGRIP1L, IRX6*
	18	25.0–27.0	3.88	*NDRG4*
RDN and MRY	1	151.5–152.0	2.89	
	3	80.5–81.0	3.01	
	15	45.5–46.0	2.90	*ZNF215*
	15	47.5–48.0	2.85	
	15	53.5–56.5	3.08	*P4HA3, KCNE3, CHRDL2, NEU3*
	15	58.5–60.0	3.54	
	15	65.5–67.0	3.22	
	21	12.5–13.0	3.18	*MCTP2*
	21	21.0–22.5	3.01	*IQGAP1*
	24	21.5–22.0	3.11	

|XPEHH| values were averaged in non-overlapping windows of 500 kb and adjacent windows were pooled

## Discussion

### Genomic inbreeding

A continuous accumulation of inbreeding within a population is unavoidable if the number of breeding animals is finite [13, 59]. The monitoring and management of inbreeding is of special concern in populations with small effective population sizes that are intrinsically susceptible to genetic drift [60, 61]. Decreased heterozygosity is accompanied by inbreeding depression in economically important traits in dairy cattle breeding [62]. This reduction of the mean phenotypic value exists for both production and functional traits [63–65]. In addition, decreasing genetic variability reduces the capacity to react to unforeseen changes in the future (e.g., production circumstances, climate, and political regulations, dissemination of diseases) [66]. Thus, proper monitoring and management of genetic diversity in cattle breeding are highly important.

In this study, patterns of ROH and levels of genomic inbreeding differed both within and across the populations studied. The large amount of long homozygous DNA segments found in Traditional Danish Red and Groningen White-Headed represents recent inbreeding and might reflect a breeding population of limited size and consequently, mating between related animals. In Traditional Danish Red, it also reflects, at least in part, a strong reliance on semen collected long ago in the Danish national gene bank. The high inbreeding of Traditional Danish Red was also shown by Zhang et al. [67] and was underlined in our study by the high average LD along with the slow LD decay observed for the Traditional Danish Red and Groningen White-Headed breeds, which points out a narrow genetic basis. Based on pedigree-analyses, it was demonstrated that recent inbreeding has more detrimental effects compared to ancestral inbreeding, especially for production traits such as milk yield [63, 68]. As evidenced by Bjelland et al. [62] and Pryce et al. [65], genomic metrics such as ROH are also suitable to quantify inbreeding depression. However, the results about whether the size of ROH is correlated with its harmfulness are rather ambiguous. While Zhang et al. [69] reported that harmful variants are more likely to be associated with short to medium-sized ROH, Doekes et al. [63] found no significant differences between shorter and longer homozygous DNA-segments in terms of inbreeding depression.

The lowest amount of inbreeding was observed in German Angler. These results are in line with findings from Addo et al. [70] who reported a low mean inbreeding coefficient for German Angler of ${F}_{ROH}$ = 0.021 for ROH longer than 4 Mb. In addition, in our study, LD decay was fastest for German Angler due to its high degree of admixture. Inbreeding of the Dutch dual-purpose Meuse-Rhine-Yssel breed was intensively studied by Eynard et al. [71]. Parameter settings for the identification of ROH in their study differed from those used here. Nevertheless, based on ROH longer than 1 Mb, they reported an ${F}_{ROH}$ of 0.07, which was in accordance to our value of 0.072 based on ROH longer than 4 Mb.

### Population structure

Population structure and genetic connectedness of the cattle breeds analyzed in our study was assessed using three complementary approaches PCA, ADMIXTURE, and TreeMix. The results of the analyses agree well with each other. All the methodologies applied indicated close relationships and a similar genetic background for Red and White Dual-Purpose and Meuse-Rhine-Yssel. In the PCA plot, a considerable proportion of the variance was shared between these breeds. Furthermore, ADMIXTURE revealed a similar genetic background for these two breeds, and the phylogenetic tree indicated low genetic drift. In addition, the pairwise F_ST_ value was lowest for this combination of breeds. It is assumed that these two dual-purpose populations originate from the same genetic source, but have been kept and managed in two distinct geographical regions. Moreover, since several years, the exchange of sires between these populations contributes to an increase in genetic similarity. Our study showed a larger influence of Red Holstein genes on Red and White Dual-Purpose than on Meuse-Rhine-Yssel. Red Holstein was frequently used in Red and White Dual-Purpose in order to improve milk yield, and Addo et al. [72] identified several Red Holstein bulls as important key ancestors of the present-day Red and White Dual-Purpose population. In many herd books, the introgression of foreign genes from other breeds is accepted to a certain degree. However, since 1970 only animals with at most 25% Red Holstein ancestry are registered in the herd book of the Red and White Dual-Purpose breed [73]. In the past, Holstein Friesian has also been massively used in the Meuse-Rhine-Yssel breed resulting in a substantial reduction of purebred individuals in The Netherlands [71]. Nowadays, the use of higher-yielding breeds in Dutch Meuse-Rhine-Yssel is more strictly limited, since sires of that breed are often used for crossbreeding in Holstein Friesian [74]. In order to generate a high degree of heterosis, efforts have been made to minimize the proportion of Holstein genes in this breed. Our results show that the Deep Red breed is genetically similar to both Red and White Dual-Purpose and Meuse-Rhine-Yssel, i.e. clustering of the three breeds in the PCA, the low estimates of F_ST_ and gene flow between Deep Red and Meuse-Rhine-Yssel revealed by the phylogenetic tree. Genetic connectedness between these breeds was already described by Marjanovic et al. [75] and van Breukelen et al. [76]. Dutch Improved Red was recently developed and derived from Meuse-Rhine-Yssel with major genetic contributions from Deep Red [76, 77]. Recent gene flow from the closely-related breeds, Meuse-Rhine-Yssel and Deep Red, to Improved Red might explain that no distinct ancestry component was detectable for Improved Red in the ADMIXTURE analysis.

Our study uncovered the highest degree of genetic differentiation for Traditional Danish Red compared to all other breeds. The PCA showed that the Traditional Danish Red breed was clearly separated from all other breeds. On the one hand, this is caused by a different geographical origin. On the other hand, the ADMIXTURE analysis confirmed a unique genetic background of Traditional Danish Red and a low level of admixture with other breeds. In addition, pairwise F_ST_ values were highest between Traditional Danish Red and all other breeds, which indicates a high degree of genetic differentiation. The unique genetic background and a low level of admixture of Traditional Danish Red were also confirmed by Gautason et al. [78]. In our study, the Groningen White-Headed breed was found to be genetically more distinct from the other Dutch cattle populations, which agrees with previous findings [76, 79]. From the 1970s onwards, crossbreeding with Holstein Friesian was reported to improve the productivity of the native Groningen White-Headed [8]. In local cattle breeds, crossbreeding with animals from economically superior breeds has been common practice for a long time [7, 80, 81]. However, today introgression of foreign genetic material is viewed with a more critical eye because, as a result, the native genetic constitution of the recipient breed is eliminated and lost [82, 83]. This is impressively demonstrated for the German Angler breed. In our study, a high degree of admixture with strong gene flow from Red Holstein to German Angler was observed, which confirms the high level of genetic heterogeneity of German Angler reported by Addo et al. [70]. The strong introgression of Red Holstein in German Angler was already described by Bennewitz and Meuwissen [84], who reported that the original genetic background of the native old-type Angler is nearly extinct.

### Signatures of selection

In order to investigate whether directional selection occurred in the Red Holstein, Red and White Dual-Purpose, and Meuse-Rhine-Yssel breeds, genome-wide SNPs were used to detect signatures of selection within and across these breeds. Signatures of selection are assumed to occur in genomic regions that host essential genes with a role in the phenotypes that were selected for [49]. For the Red Holstein breed, the strongest signatures of selection detected were located on BTA18, which is known to harbor many quantitative trait loci (QTL) that affect various traits in dairy cattle such as reproduction, calving traits, somatic cell count and conformation traits [85–88]. For instance, in a genome-wide association study, Müller et al. [89] identified a QTL on BTA18 (17.5 Mb) linked to maternal stillbirth in Holstein Friesian. Moreover, they found a nearby QTL (BTA18 at 17.1 Mb) that affects days open and days from calving to first insemination. In our study, putative signatures of selection were detected on BTA18 (18.5–19.0 Mb), which carries the *BRD7* gene and was previously described as associated with protein yield in Holstein cattle [90, 91]. In addition, a signature of selection was located in close proximity to the *FTO* gene (22.0–22.5 Mb). Zielke et al. [92] identified SNPs that are located in this genomic region and are significantly associated with milk fat yield in German Holstein cattle. Basically, the strong signatures of selection detected in Red Holstein on BTA18 in our study are indicative of directed selection on production traits. In Red and White Dual-Purpose, signatures of positive selection were found on six chromosomes. On BTA9, *BACH2* was identified as a key gene involved in the metabolism of milk fatty acids in dairy cattle [93]. Recently, Chen et al. [94] reported a SNP that is significantly associated with milking temperament in Holstein cattle in close proximity to the *RARS2*, *SLC35A1* and *CFAP206* genes. On BTA15, the *EIF4G2* gene was located within a genomic region that influences lactation persistency in a genome-wide association study conducted by Do et al. [95]. Moreover, also on BTA15, *SBF2* was in close proximity to a SNP linked to growth traits in Charolais cattle [96]. On BTA20, the detected signatures of selection were located close to each other and flanked the *RAI14* and *ADAMTS12* genes. *RAI14* is located close to genomic regions associated with susceptibility of clinical mastitis in Holstein Friesian [97] and milking speed in Scandinavian Holsteins [98]. The *ADAMTS12* gene has been reported in various genome-wide association studies for traits such as carcass weight and milk production [99–101]. In Meuse-Rhine-Yssel, signatures of selection were located on BTA1, 3 and 15. The *GRAMD1B* gene that flanks the genomic region around the signature of selection at 34.3 Mb on BTA15 is associated with female fertility in Nordic Red cattle [102]. Furthermore, Cole et al. [87] detected in the same genomic region (34.8 Mb, BTA15) a significant signature of selection, for the conformation trait, rear teat placement.

The XPEHH analysis of Red Holstein and Red and White Dual-Purpose showed directional selection pressure in Red Holstein in the genomic region between 14.0 and 15.5 Mb on BTA18. Similarly, in Red Holstein, Rothammer et al. [103] reported signatures of selection in this particular genomic region and pointed out the proximity to the *MC1R* gene, which is responsible for variation in red coat color [104]. Red Holstein has been subjected to selective breeding on red coat color in order to maintain the desired coat color-phenotype, which might differentiate this breed from Red and White Dual-Purpose. Putative signatures of selection in the genomic region between 16.0 and 24.0 Mb on BTA18 were detected in the two comparisons of Red Holstein with Red and White Dual-Purpose and of Red Holstein with Meuse-Rhine-Yssel. As described above, genes in these regions (e.g., *BRD7* and *FTO*) are mainly associated with milk production traits and our results suggest directed selection for these traits in Red Holstein. The comparison of Red Holstein with Meuse-Rhine-Yssel revealed a high $|\mathrm{XPEHH}|$ score on BTA18 in the genomic region between 15.0 and 15.5 Mb, which encompasses the *VPS35* gene. In Swedish Red cattle, Duchemin et al. [105] found an intronic SNP in the *VPS35* gene that is associated with non-coagulating milk. Furthermore, the study of Pimentel et al. [106] showed a SNP located in the *NDRG4* gene (BTA18 at 26.3 Mb) that is significantly associated with fat and protein yield, interval from calving to first insemination, and days open in cows. XPEHH-analysis between Red and White Dual-Purpose and Meuse-Rhine-Yssel revealed a set of putative signals on BTA15 between 45.5 and 67.0 Mb. A genome-wide association study in Holstein cattle discovered a significant SNP for milk yield located in the *P4HA3* gene at 54.4 Mb on BTA15 [107]. Moreover, Doyle et al. [108] reported suggestive SNPs for beef traits that are located in the *CHRDL2* and *NEU3* genes on BTA15. Beyond that, *NEU3* was demonstrated to have functional effects on fertility and production traits in Holstein Friesian [109]. In our study, other signatures of selection were found on BTA21 that harbors the *MCTP2* gene, which was previously reported as a candidate gene for traits related to carcass merit and metabolic weight in different cattle breeds [110, 111]. The XPEHH analysis of Red and White Dual-Purpose and Meuse-Rhine-Yssel also revealed a signal on BTA21 between 21.0 and 22.5 Mb, which encompasses the *IQGAP2* gene that is strongly associated with sole hemorrhage in Holstein cattle [112].

### Implications

In this study, different population genetic analyses were used to investigate the population structure and connectedness of certain red cattle breeds from Northern Europe at the genomic level. Ultimately, the objective is to create a multi-breed reference population in order to ensure a sustainable development and the conservation of these native breeds. Our results provide initial evidence that some of the red cattle populations studied (e.g., Red and White Dual-Purpose, Meuse-Rhine-Yssel, and Deep Red) might benefit from a common prospective multi-breed genomic evaluation provided that the number of animals included in the training population is sufficiently large [113]. However, implementation of a genomic evaluation for small-sized populations is an ambitious venture and faces a number of challenges. The key element of genomic prediction is LD, i.e., the non-random association between genetic markers and causal variants. Thus, the utility of genomic sites for the estimation of GEBV depends on the extent of LD with the QTL. The stronger the LD, the more accurately will GEBV be predicted [114], and such LD is known to be breed-specific [115, 116]. Consequently, marker effects are not consistent among more distantly related populations due to differences in LD. As successfully shown in several studies [17, 18, 113], the combination of divergent cattle populations can increase the predictive ability of GEBV, to some degree, depending on their relatedness. However, further studies must explore to what extent genomic prediction can work across red cattle breeds in Northern European countries.

## Conclusions

This study represents a detailed genetic characterization of ten cattle breeds belonging to the Red group in Northern Europe. The results indicate that some breeds are genomically distinct (e.g., Traditional Danish Red and Groningen White-Headed), whereas other populations show strong genetic similarity (e.g., Red and White Dual-Purpose, Meuse-Rhine-Yssel, and Deep Red). Furthermore, we show that some breeds (e.g., German Angler and Red and White Dual-Purpose) have experienced intense gene flow in the past from higher yielding breeds such as Red Holstein. The results are relevant for application by breeding organizations and breed associations in order to guide and develop prospective breeding strategies.

## Supplementary Information

**Additional file 1: Table S1.** Information on breeds, sample size per breed, source and type of SNP chip.

**Additional file 2: Table S2.** Details on data processing. For each analysis, steps of quality control and the size of used dataset are specified.

**Additional file 3: Table S3.** Descriptive statistics of frequency and size of ROH per breed.

**Additional file 4: Table S4.** Average level of genomic inbreeding (F_ROH_) for different length categories with standard deviation in parenthesis per breed.

**Additional file 5: Figure S1.** Linkage disequilibrium (LD) decay across the genome as a function of inter-marker distance for each breed. ANG: German Angler. DBE: Dutch Belted. DFR: Dutch Friesian Red. DR: Deep Red. GWH: Groningen White-Headed. IR: Improved Red. MRY: Meuse-Rhine-Yssel. RDM70: Traditional Danish Red. RDN: Red and White Dual-Purpose. RH: Red Holstein.

**Additional file 6**:** Figure S2.** Genetic relatedness among the red cattle breeds from Germany, The Netherlands and Denmark (circles) and further European cattle breeds from the WIDDE database (triangles) using principle component analyses (a: PC1 vs. PC2; b: PC2 vs. PC3; c: PC2 vs. PC4). ANG: German Angler, AYR: Finnish Ayrshire, BRV: Braunvieh, BSW: Brown Swiss, DBE: Dutch Belted, DFR: Dutch Friesian Red, DR: Deep Red, GNS: Guernsey, GWH: Groningen White-Headed, HOL: Holstein Friesian, IR: Improved Red, JER: Jersey, MON: Montbeliarde, MRY: Meuse-Rhine-Yssel, NRC: Norwegian Red Cattle, PRP: French Red Pied Lowland, RDM70: Traditional Danish Red, RDN: Red and White Dual-Purpose, RH: Red Holstein, SHO: Shorthorn, SIM: Simmental.

**Additional file 7: Figure S3.** Plot of ADMIXTURE cross validation error from K = 2 to K = 40 revealed lowest cross validation error at K = 8.

**Additional file 8: Figure S4.** Unsupervised model-based clustering results of 393 individuals using 19,717 SNPs. Presented is K = 2 to K = 12. ANG: German Angler. DBE: Dutch Belted. DFR: Dutch Friesian Red. DR: Deep Red. GWH: Groningen White-Headed. IR: Improved Red. MRY: Meuse-Rhine-Yssel. RDM70: Traditional Danish Red. RDN: Red and White Dual-Purpose. RH: Red Holstein.

**Additional file 9: Figure S5.** Unsupervised model-based clustering results of 653 individuals from 21 breed. Presented is K = 10 to K = 18, with optimal number of ancestral populations K = 18. ANG: German Angler, AYR: Finnish Ayrshire, BRV: Braunvieh, BSW: Brown Swiss, DBE: Dutch Belted, DFR: Dutch Friesian Red, DR: Deep Red, GNS: Guernsey, GWH: Groningen White-Headed, HOL: Holstein Friesian, IR: Improved Red, JER: Jersey, MON: Montbeliarde, MRY: Meuse-Rhine-Yssel, NRC: Norwegian Red Cattle, PRP: French Red Pied Lowland, RDM70: Traditional Danish Red, RDN: Red and White Dual-Purpose, RH: Red Holstein, SHO: Shorthorn, SIM: Simmental.

**Additional file 10: Figure S6.** Plot of f-index, representing the fraction of the variance in the sample covariance matrix explained by the model covariance matrix, as criteria for model fitting for number of migration events from 0 to 10.

**Additional file 11: Table S5.** Negative z-scores obtained from the f3-statistics in THREEPOP.

**Additional file 12: Table S6.** Pairwise genome-wide F_ST_ values (based on Weir and Cockerham [48]) for all combinations of studied breeds.

**Additional file 13: Table S7.** Full list of annotated genes located in genomic regions detected as putative selection signatures.

## Data Availability

The datasets of the German breeds used and analysed during the current study are owned by Rinderzucht Schleswig–Holstein (RSH e.G.) and were provided via a signed data access agreement which does not allow for data sharing. Interested, qualified researchers may request these data by contacting RSH eG at rsheg@rsheg.de. The data of Dutch and Danish cattle breeds are not publicly available. The data from the WIDDE database are publicly available at http://widde.toulouse.inra.fr/widde/.
