# Supplementary material for: Assessing the genetic background and genomic relatedness of red cattle populations originating from Northern Europe
Source: Genet Sel Evol. 2021 Mar 6;53:23. doi: 10.1186/s12711-021-00613-6 (PMC7936461; doi:10.1186/s12711-021-00613-6)
Supplement: Supplementary file 1 — Additional file 1: Table S1. Information on breeds, sample size per breed, source and type of SNP chip. [file 12711_2021_613_MOESM1_ESM.docx]

**Additional file 1 Table S1**

**Table S1** Information on breeds, sample size per breed, source and type of SNP chip

| **Breed** | **Breed code** | **Number of samples** | **Source** | **Chip type** |
| --- | --- | --- | --- | --- |
| Red and White Dual-Purpose | RDN | 744/50^a^ | RSH eG^c^ | Illumina BovineSNP50v3 |
| German Angler | ANG | 106/50 ^a^ | RSH eG | Illumina BovineSNP50v1 |
| Red Holstein | RH | 88/50 ^a^ | RSH eG | Illumina BovineSNP50v1 |
| Meuse-Rhine-Yssel | MRY | 292/50 ^a^ | CGN^d^ | Illumina BovineSNP50v2/v3,  Illumina BovineHD |
| Deep Red | DR | 21 | CGN | Illumina BovineSNP50v2 |
| Dutch Friesian Red | DFR | 51/50 ^a^ | CGN | Illumina BovineSNP50v3 |
| Groningen White-Headed | GWH | 36 | CGN | Illumina BovineSNP50v3 |
| Dutch Belted | DBE | 16 | CGN | Illumina BovineSNP50v2/v3 |
| Improved Red | IR | 21 | CGN | Illumina BovineSNP50v3 |
| Traditional Danish Red | RDM70 | 50 | Aarhus University | Illumina BovineHD |
| Finnish Ayrshire | AYR^1^ | 18^b^ | WIDDE^e^ | Illumina BovineSNP50v2 |
| Holstein Friesian | HOL^2^ | 32 ^b^ | WIDDE | Illumina BovineSNP50v1 |
| N´Dama | NDA^2^ | 25 ^b^ | WIDDE | Illumina BovineSNP50v1 |
| Norwegian Red Cattle | NRC^2^ | 21 ^b^ | WIDDE | Illumina BovineSNP50v1 |
| French Red Pied Lowland | PRP^3^ | 22 ^b^ | WIDDE | Illumina BovineSNP50v1 |
| Shorthorn | SHO^1^ | 24 ^b^ | WIDDE | Illumina BovineSNP50v2 |
| Guernsey | GNS^2^ | 21 ^b^ | WIDDE | Illumina BovineSNP50v1 |
| Jersey | JER^2^ | 28 ^b^ | WIDDE | Illumina BovineSNP50v1 |
| Braunvieh | BRV^1^ | 20 ^b^ | WIDDE | Illumina BovineSNP50v2 |
| Brown Swiss | BSW^2^ | 24 ^b^ | WIDDE | Illumina BovineSNP50v1 |
| Montbeliarde | MON^3^ | 30 ^b^ | WIDDE | Illumina BovineSNP50v1 |
| Simmental | SIM^4^ | 20 ^b^ | WIDDE | Illumina BovineSNP50v1 |

^a^ 50 randomly sampled animals for the PCA, ADMIXTURE and TreeMix

^b^ Samples from WIDDE have only been used for the TreeMix analysis

^c^ Rinderzucht Schleswig-Holstein e.G.

^d^ Center for Genetic Resources, the Netherlands

^e^ Web-Interfaced next generation database for genetic diversity exploration

^1^ Decker et al. [43]; ^2^Matukumalli et al [41]; ^3^Gautier et al. [42]; ^4^Gao et al. [44]
