# Supplementary material for: Assessing the genetic background and genomic relatedness of red cattle populations originating from Northern Europe
Source: Genet Sel Evol. 2021 Mar 6;53:23. doi: 10.1186/s12711-021-00613-6 (PMC7936461; doi:10.1186/s12711-021-00613-6)
Supplement: Supplementary file 2 — Additional file 2: Table S2. Details on data processing. For each analysis, steps of quality control and the size of used dataset are specified. [file 12711_2021_613_MOESM2_ESM.docx]

**Additional file 2 Table S2**

**Table S2** Details on data processing. For each analysis, steps of quality control and the size of used dataset are specified.

|  | Quality control | | Size of dataset | | | |
| --- | --- | --- | --- | --- | --- | --- |
| Analysis | Applied filters | LD thinning | Number of breeds | Total number of animals | Animals per breed | Number of SNPs |
| Observed and expected heterozygosity | --mind 0.1  --geno 0.1 |  | 10 | 1,425 | 16-744 | 36,195 |
| Runs of homozygosity | --mind 0.1  --geno 0.1 |  | 10 | 1,425 | 16-744 | 36,195 |
| LD estimation | --mind 0.1  --geno 0.1 |  | 10 | 1,425 | 16-744 | 36,195 |
| Principal Component Analysis | --mind 0.1  --geno 0.1 |  | 10 | 394 | 16-50^a^ | 36,195 |
| ADMIXTURE | --mind 0.1  --geno 0.1 | --bp-space option | 10 | 393^b^ | 15-50^a^ | 19,717 |
| TreeMix | --mind 0.1  --geno 0.1 | --bp-space option | 22 | 678^b^ | 15-50^a^ | 19,294 |
| F_ST_ values | --mind 0.1  --geno 0.1 |  | 22 | 678 | 15-50 | 35,101 |
| Selection signatures | --mind 0.1  --geno 0.1 |  | 3 | 1,124 | 88-744 | 36,195 |

^a^Up to 50 randomly sampled animals for PCA, ADMIXTURE and TreeMix

^b^One individual of Dutch Belted was excluded from the analysis ADMIXTURE and TreeMix due to high proportion of admixed ancestry.
