# Supplementary material for: Assessing the genetic background and genomic relatedness of red cattle populations originating from Northern Europe
Source: Genet Sel Evol. 2021 Mar 6;53:23. doi: 10.1186/s12711-021-00613-6 (PMC7936461; doi:10.1186/s12711-021-00613-6)
Supplement: Supplementary file 3 — Additional file 3: Table S3. Descriptive statistics of frequency and size of ROH per breed. [file 12711_2021_613_MOESM3_ESM.docx]

**Table S3** Descriptive statistics of frequency and size of ROH per breed

|  |  | **Number of ROH per breed** | | | **Length of ROH per breed (Mb)** | | |
| --- | --- | --- | --- | --- | --- | --- | --- |
|  |  | ROH length category | | | ROH length category | | |
| Breed | Statistic | >4 Mb | >8 Mb | >16 Mb | >4 Mb | >8 Mb | >16 Mb |
| ANG  (n = 106) | Mean  SD  Min | 7.7 | 3.5 | 1.6 | 69.9 | 51.2 | 42.2 |
|  |  | 4.1 | 2.4 | 0.9 | 44.3 | 37.8 | 28.1 |
|  |  | 1 | 1 | 1 | 6.1 | 8.0 | 16.2 |
|  | Max | 19 | 12 | 5 | 201.4 | 175.5 | 133.1 |
| DBE  (n = 16) | Mean  SD  Min  Max | 23.1 | 11.4 | 4.1 | 239.5 | 178.5 | 103.8 |
|  |  | 6.2 | 4.4 | 2.8 | 89.7 | 84.2 | 79.1 |
|  |  | 14 | 5 | 1 | 119.2 | 60.9 | 16.7 |
|  |  | 36 | 21 | 12 | 443.4 | 377.3 | 348.3 |
| DFR  (n = 51) | Mean  SD  Min  Max | 18.2 | 7.9 | 3.2 | 171.3 | 117.9 | 80.1 |
|  |  | 8.5 | 5.1 | 2.1 | 98.3 | 84.3 | 59.4 |
|  |  | 7 | 1 | 1 | 35.8 | 8.88 | 17.3 |
|  |  | 50 | 23 | 8 | 495.7 | 348.4 | 235.8 |
| DR  (n = 21) | Mean  SD  Min  Max | 14.5 | 6.4 | 2.8 | 133.2 | 93.2 | 70.5 |
|  |  | 7.2 | 4.4 | 2.0 | 84.3 | 74.8 | 51.2 |
|  |  | 5 | 1 | 1 | 30.0 | 10.0 | 16.8 |
|  |  | 31 | 15 | 7 | 331.4 | 244.3 | 170.7 |
| GWH  (n = 36) | Mean  SD  Min  Max | 35.3 | 15.7 | 4.8 | 327.1 | 225.5 | 115.7 |
|  |  | 7.8 | 5.4 | 2.8 | 101.7 | 96.3 | 75.1 |
|  |  | 19 | 5 | 1 | 148.2 | 45.0 | 18.9 |
|  |  | 50 | 26 | 11 | 574.9 | 439.7 | 287.9 |
| IR  (n = 21) | Mean  SD  Min  Max | 7.3 | 4.6 | 4.2 | 79.8 | 80.2 | 138.3 |
|  |  | 6.3 | 4.6 | 3.7 | 116.0 | 120.2 | 148.2 |
|  |  | 1 | 1 | 1 | 5.7 | 8.7 | 28.0 |
|  |  | 23 | 15 | 11 | 503.5 | 462.9 | 417.3 |
| MRY  (n = 292) | Mean  SD  Min  Max | 17.9 | 8.2 | 3.2 | 179.3 | 128.6 | 82.6 |
|  |  | 7.1 | 4.0 | 2.0 | 88.5 | 77.6 | 62.7 |
|  |  | 2 | 1 | 1 | 12.2 | 8.0 | 16.0 |
|  |  | 42 | 28 | 12 | 587.0 | 523.4 | 451.4 |
| RDM70  (n = 50) | Mean  SD  Min  Max | 40.8 | 17.8 | 5.4 | 388.3 | 264.0 | 135.3 |
|  |  | 9.0 | 5.4 | 3.7 | 143.2 | 134.2 | 125.8 |
|  |  | 24 | 7 | 1 | 171.6 | 79.9 | 16.4 |
|  |  | 74 | 41 | 24 | 1105.2 | 981.7 | 826.9 |
| RDN  (n = 744) | Mean  SD  Min  Max | 12.3 | 5.4 | 2.3 | 120.6 | 83.7 | 58.9 |
|  |  | 5.6 | 3.3 | 1.6 | 71.8 | 62.7 | 50.0 |
|  |  | 1 | 1 | 1 | 6.5 | 8.1 | 16.1 |
|  |  | 49 | 23 | 14 | 580.9 | 554.7 | 425.4 |
| RH  (n = 88) | Mean  SD  Min  Max | 15.6 | 7.2 | 2.8 | 156.7 | 109.9 | 68.6 |
|  |  | 5.2 | 3.4 | 1.6 | 64.3 | 57.9 | 44.3 |
|  |  | 4 | 1 | 1 | 34.4 | 8.2 | 16.1 |
|  |  | 28 | 17 | 8 | 317.6 | 273.9 | 238.2 |
