# Supplementary material for: Assessing the genetic background and genomic relatedness of red cattle populations originating from Northern Europe
Source: Genet Sel Evol. 2021 Mar 6;53:23. doi: 10.1186/s12711-021-00613-6 (PMC7936461; doi:10.1186/s12711-021-00613-6)
Supplement: Supplementary file 4 — Additional file 4: Table S4. Average level of genomic inbreeding (FROH) for different length categories with standard deviation in parenthesis per breed. [file 12711_2021_613_MOESM4_ESM.docx]

**Table S4** Average level of genomic inbreeding (F_ROH_) for different length categories with standard deviation in parenthesis per breed.

| Breed | F_ROH>4 Mb_ | F_ROH>8 Mb_ | F_ROH>16 Mb_ |
| --- | --- | --- | --- |
| ANG | 0.028 (0.018) | 0.021 (0.015) | 0.017 (0.011) |
| DBE | 0.096 (0.040) | 0.071 (0.034) | 0.042 (0.032) |
| DFR | 0.069 (0.041) | 0.047 (0.024) | 0.032 (0.024) |
| DR | 0.053 (0.034) | 0.037 (0.030) | 0.028 (0.021) |
| GWH | 0.131 (0.041) | 0.090 (0.039) | 0.046 (0.030) |
| IR | 0.032 (0.046) | 0.032 (0.048) | 0.053 (0.059) |
| MRY | 0.072 (0.035) | 0.052 (0.031) | 0.033 (0.025) |
| RDM70 | 0.155 (0.057) | 0.106 (0.054) | 0.052 (0.050) |
| RDN | 0.048 (0.029) | 0.033 (0.025) | 0.024 (0.020) |
| RH | 0.063 (0.026) | 0.044 (0.023) | 0.027 (0.018) |
