# Supplementary figures and images for: Assessing the genetic background and genomic relatedness of red cattle populations originating from Northern Europe
Source: Genet Sel Evol. 2021 Mar 6;53:23. doi: 10.1186/s12711-021-00613-6 (PMC7936461; doi:10.1186/s12711-021-00613-6)

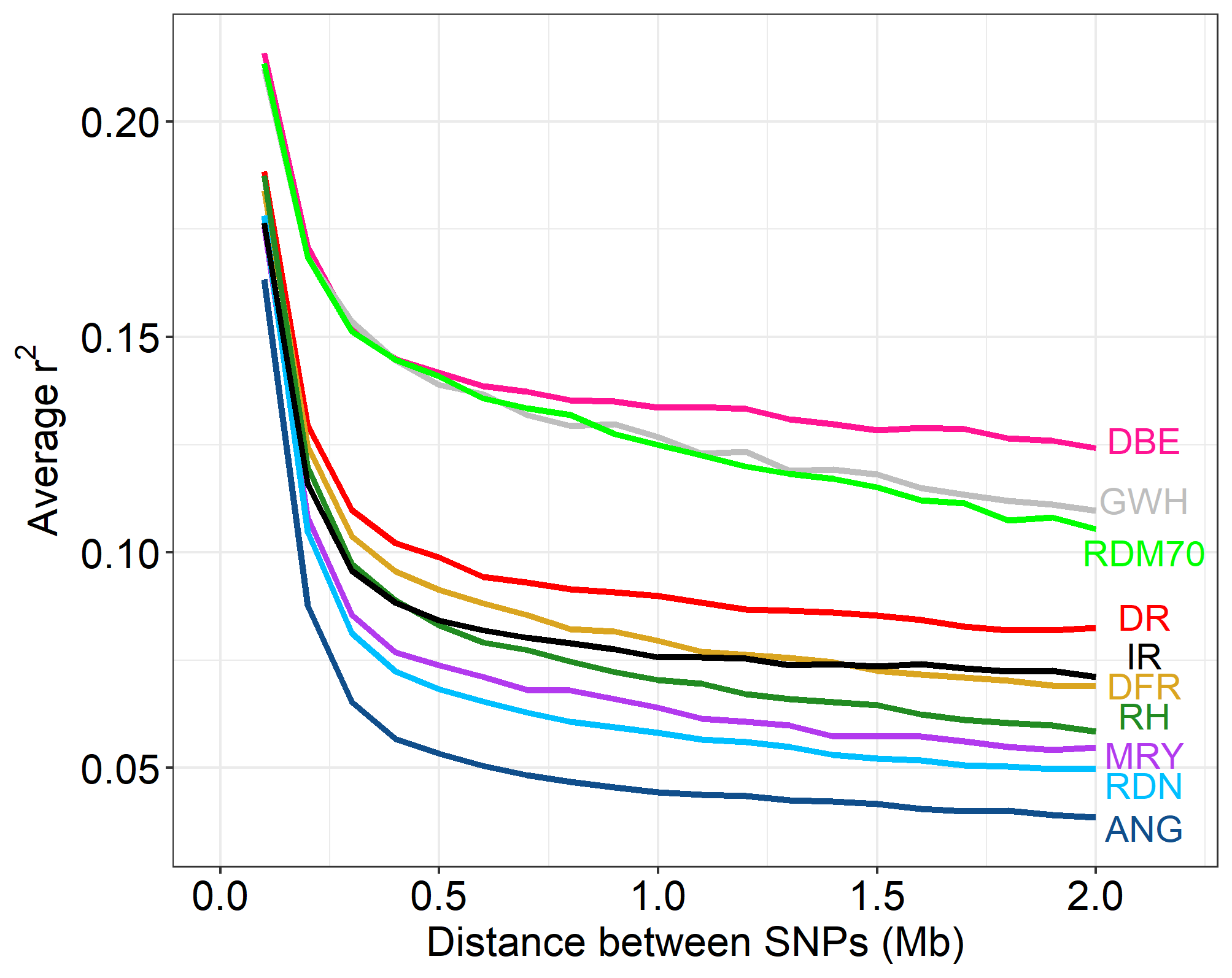

Supplement: Supplementary file 5 — Additional file 5: Figure S1. Linkage disequilibrium (LD) decay across the genome as a function of inter-marker distance for each breed. ANG: German Angler. DBE: Dutch Belted. DFR: Dutch Friesian Red. DR: Deep Red. GWH: Groningen White-Headed. IR: Improved Red. MRY: Meuse-Rhine-Yssel. RDM70: Traditional Danish Red. RDN: Red and White Dual-Purpose. RH: Red Holstein. [file 12711_2021_613_MOESM5_ESM.tiff]

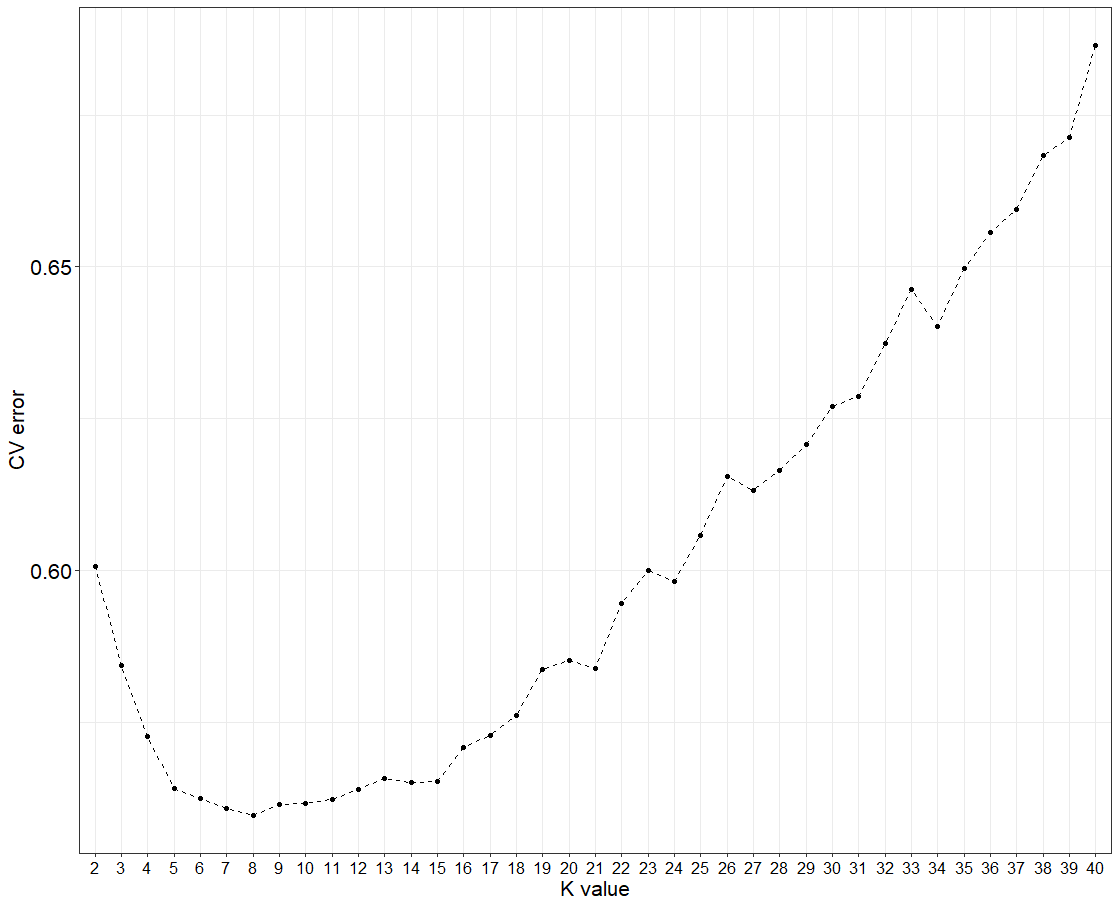

Supplement: Supplementary file 7 — Additional file 7: Figure S3. Plot of ADMIXTURE cross validation error from K = 2 to K = 40 revealed lowest cross validation error at K = 8. [file 12711_2021_613_MOESM7_ESM.png]

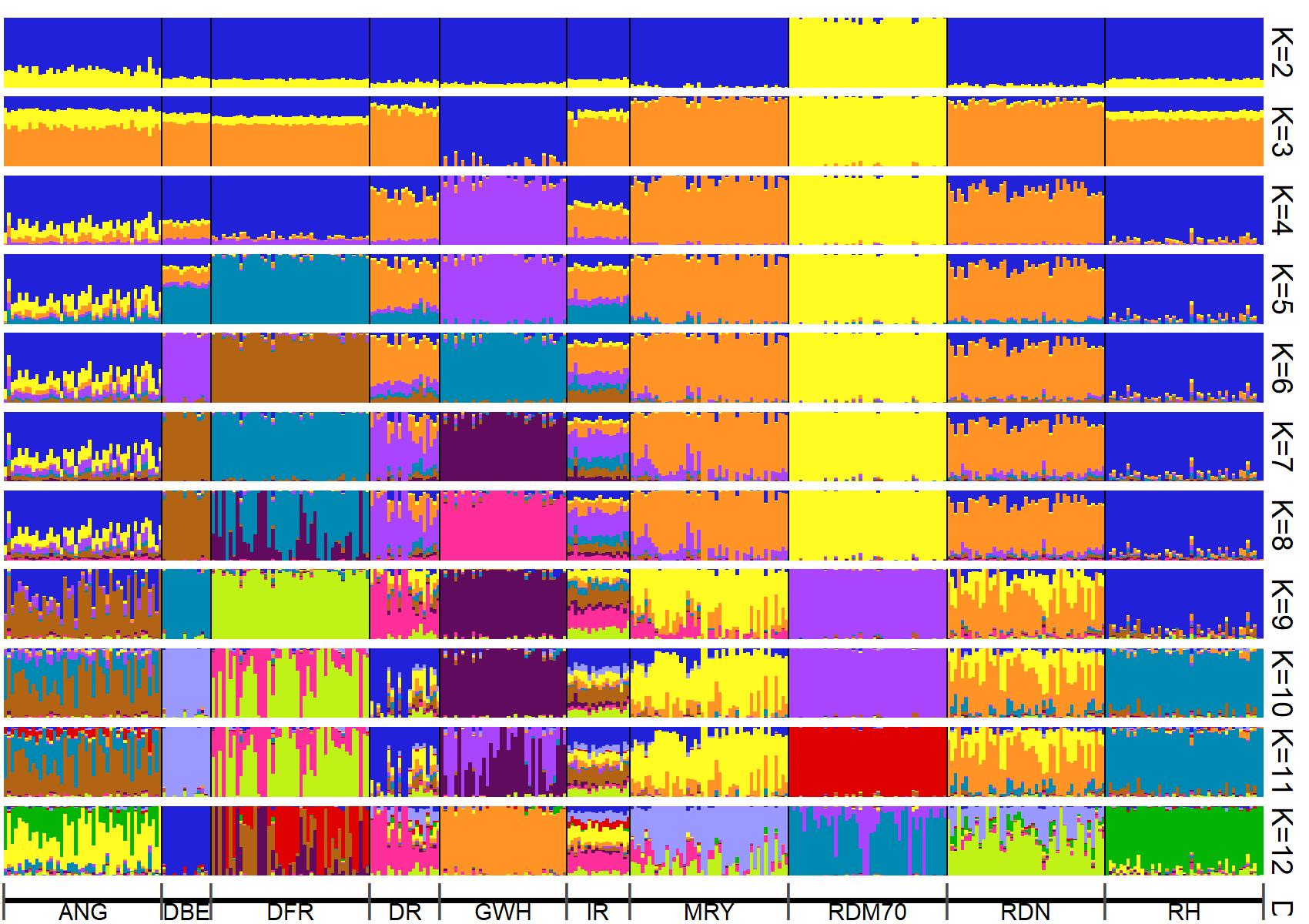

Supplement: Supplementary file 8 — Additional file 8: Figure S4. Unsupervised model-based clustering results of 393 individuals using 19,717 SNPs. Presented is K = 2 to K = 12. ANG: German Angler. DBE: Dutch Belted. DFR: Dutch Friesian Red. DR: Deep Red. GWH: Groningen White-Headed. IR: Improved Red. MRY: Meuse-Rhine-Yssel. RDM70: Traditional Danish Red. RDN: Red and White Dual-Purpose. RH: Red Holstein. [file 12711_2021_613_MOESM8_ESM.jpg]

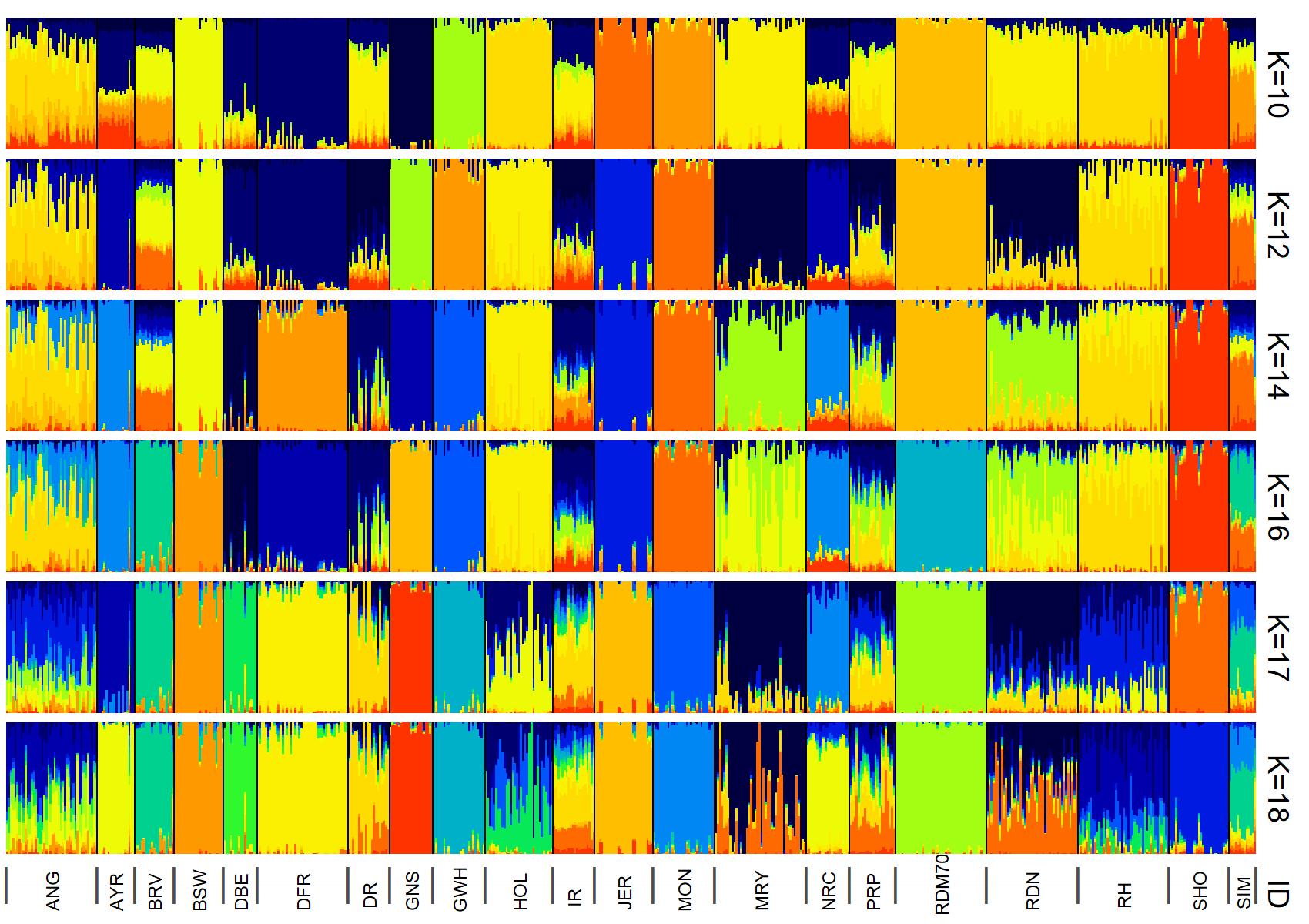

Supplement: Supplementary file 9 — Additional file 9: Figure S5. Unsupervised model-based clustering results of 653 individuals from 21 breed. Presented is K = 10 to K = 18, with optimal number of ancestral populations K = 18. ANG: German Angler, AYR: Finnish Ayrshire, BRV: Braunvieh, BSW: Brown Swiss, DBE: Dutch Belted, DFR: Dutch Friesian Red, DR: Deep Red, GNS: Guernsey, GWH: Groningen White-Headed, HOL: Holstein Friesian, IR: Improved Red, JER: Jersey, MON: Montbeliarde, MRY: Meuse-Rhine-Yssel, NRC: Norwegian Red Cattle, PRP: French Red Pied Lowland, RDM70: Traditional Danish Red, RDN: Red and White Dual-Purpose, RH: Red Holstein, SHO: Shorthorn, SIM: Simmental. [file 12711_2021_613_MOESM9_ESM.jpg]

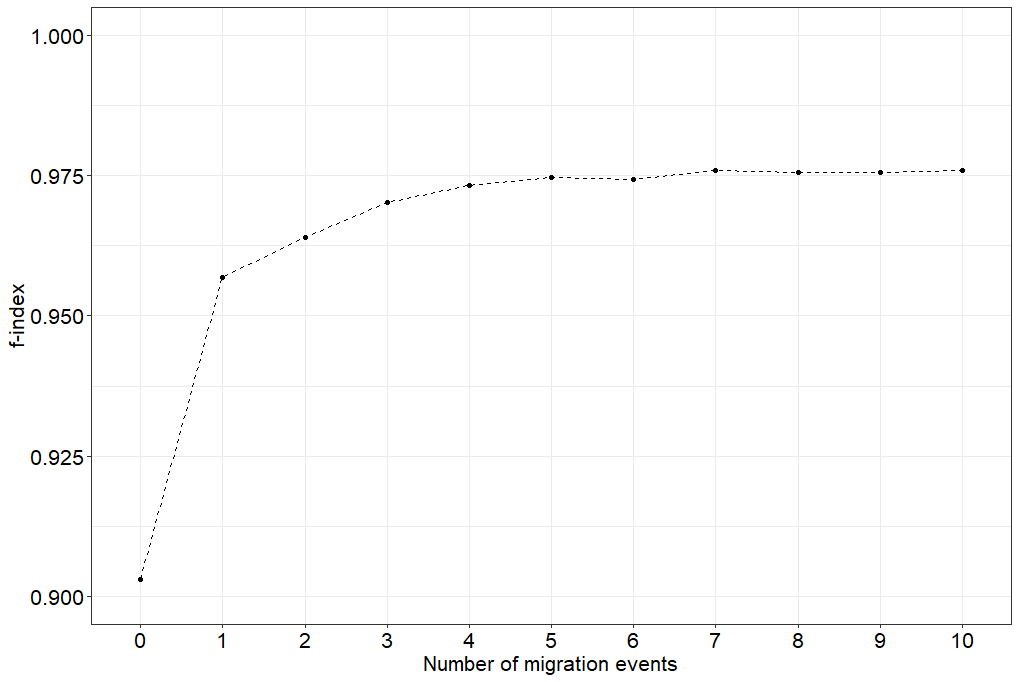

Supplement: Supplementary file 10 — Additional file 10: Figure S6. Plot of f-index, representing the fraction of the variance in the sample covariance matrix explained by the model covariance matrix, as criteria for model fitting for number of migration events from 0 to 10. [file 12711_2021_613_MOESM10_ESM.png]
