# Supplementary material for: Assessing the genetic background and genomic relatedness of red cattle populations originating from Northern Europe
Source: Genet Sel Evol. 2021 Mar 6;53:23. doi: 10.1186/s12711-021-00613-6 (PMC7936461; doi:10.1186/s12711-021-00613-6)
Supplement: Supplementary file 6 — Additional file 6: Figure S2. Genetic relatedness among the red cattle breeds from Germany, The Netherlands and Denmark (circles) and further European cattle breeds from the WIDDE database (triangles) using principle component analyses (a: PC1 vs. PC2; b: PC2 vs. PC3; c: PC2 vs. PC4). ANG: German Angler, AYR: Finnish Ayrshire, BRV: Braunvieh, BSW: Brown Swiss, DBE: Dutch Belted, DFR: Dutch Friesian Red, DR: Deep Red, GNS: Guernsey, GWH: Groningen White-Headed, HOL: Holstein Friesian, IR: Improved Red, JER: Jersey, MON: Montbeliarde, MRY: Meuse-Rhine-Yssel, NRC: Norwegian Red Cattle, PRP: French Red Pied Lowland, RDM70: Traditional Danish Red, RDN: Red and White Dual-Purpose, RH: Red Holstein, SHO: Shorthorn, SIM: Simmental. [file 12711_2021_613_MOESM6_ESM.pdf]

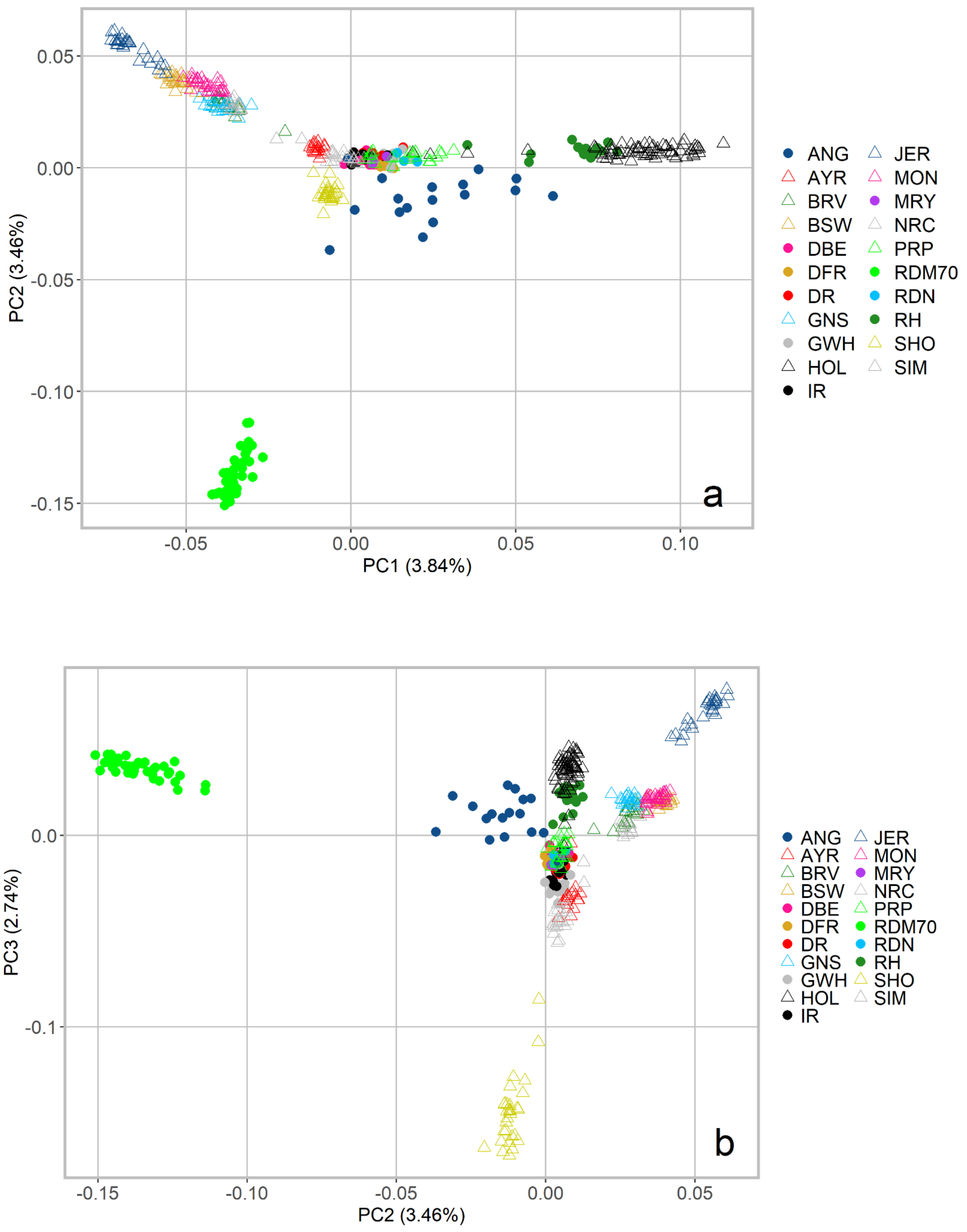
a
PC1 (3.84%)
PC2 (3.46%)
b
PC2 (3.46%)
PC3 (2.74%)
ANG
AYR
BRV
BSW
DBE
DFR
DR
GNS
GWH
HOL
IR
JER
MON
MRY
NRC
PRP
RDM70
RDN
RH
SHO
SIM

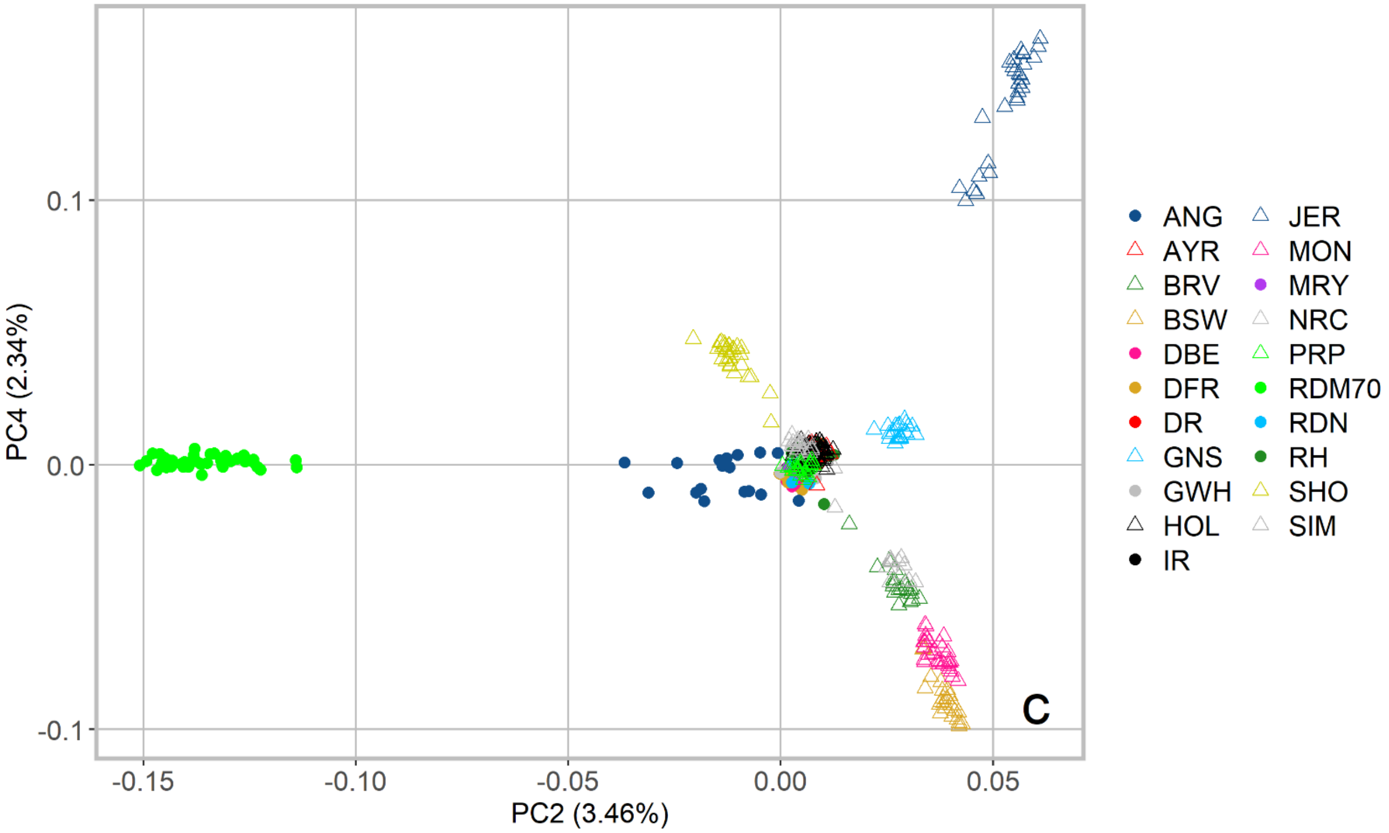

Figure S2 Genetic relatedness among the red cattle breeds from Germany, the Netherlands and Denmark (circles) and further European cattle breeds from the WIDDE database (triangles) using principle component analyses (a: PC1 vs. PC2; b: PC2 vs. PC3; c: PC2 vs. PC4). ANG: German Angler, AYR: Finnish Ayrshire, BRV: Braunvieh, BSW: Brown Swiss, DBE: Dutch Belted, DFR: Dutch Friesian Red, DR: Deep Red, GNS: Guernsey, GWH: Groningen White-Headed, HOL: Holstein Friesian, IR: Improved Red, JER: Jersey, MON: Montbeliarde, MRY: Meuse-Rhine-Yssel, NRC: Norwegian Red Cattle, PRP: French Red Pied Lowland, RDM70: Traditional Danish Red, RDN: Red and White Dual-Purpose, RH: Red Holstein, SHO: Shorthorn, SIM: Simmental.
