# Supplementary material for: Assessing the genetic background and genomic relatedness of red cattle populations originating from Northern Europe
Source: Genet Sel Evol. 2021 Mar 6;53:23. doi: 10.1186/s12711-021-00613-6 (PMC7936461; doi:10.1186/s12711-021-00613-6)
Supplement: Supplementary file 12 — Additional file 12: Table S6. Pairwise genome-wide FST values (based on Weir and Cockerham [48]) for all combinations of studied breeds. [file 12711_2021_613_MOESM12_ESM.docx]

**Table S6** Pairwise genome-wide F_ST_ values (based on Weir and Cockerham [48]) for all combinations of studied breeds.

|  | AYR | BRV | BSW | DBE | DFR | DR | GNS | GWH | HOL | IR | JER | MON | MRY | NRC | PRP | RDM70 | RDN | RH | SHO | SIM |
| --- | --- | --- | --- | --- | --- | --- | --- | --- | --- | --- | --- | --- | --- | --- | --- | --- | --- | --- | --- | --- |
| ANG | 0.047 | 0.060 | 0.088 | 0.060 | 0.057 | 0.047 | 0.084 | 0.089 | 0.034 | 0.036 | 0.105 | 0.077 | 0.068 | 0.046 | 0.026 | 0.083 | 0.053 | 0.021 | 0.088 | 0.058 |
| AYR |  | 0.083 | 0.121 | 0.087 | 0.085 | 0.072 | 0.109 | 0.123 | 0.089 | 0.061 | 0.135 | 0.103 | 0.097 | 0.041 | 0.059 | 0.136 | 0.083 | 0.083 | 0.111 | 0.078 |
| BRV |  |  | 0.080 | 0.087 | 0.087 | 0.073 | 0.096 | 0.124 | 0.090 | 0.063 | 0.123 | 0.074 | 0.097 | 0.076 | 0.060 | 0.133 | 0.083 | 0.083 | 0.121 | 0.054 |
| BSW |  |  |  | 0.127 | 0.119 | 0.111 | 0.131 | 0.152 | 0.120 | 0.104 | 0.151 | 0.109 | 0.122 | 0.114 | 0.101 | 0.158 | 0.110 | 0.114 | 0.149 | 0.100 |
| DBE |  |  |  |  | 0.067 | 0.066 | 0.116 | 0.120 | 0.084 | 0.054 | 0.145 | 0.109 | 0.087 | 0.078 | 0.054 | 0.139 | 0.073 | 0.077 | 0.124 | 0.086 |
| DFR |  |  |  |  |  | 0.065 | 0.111 | 0.106 | 0.079 | 0.053 | 0.132 | 0.102 | 0.084 | 0.074 | 0.055 | 0.122 | 0.071 | 0.072 | 0.117 | 0.087 |
| DR |  |  |  |  |  |  | 0.102 | 0.103 | 0.070 | 0.024 | 0.128 | 0.094 | 0.034 | 0.063 | 0.018 | 0.123 | 0.032 | 0.063 | 0.107 | 0.068 |
| GNS |  |  |  |  |  |  |  | 0.145 | 0.112 | 0.091 | 0.134 | 0.114 | 0.116 | 0.104 | 0.089 | 0.153 | 0.104 | 0.105 | 0.143 | 0.099 |
| GWH |  |  |  |  |  |  |  |  | 0.109 | 0.093 | 0.163 | 0.135 | 0.111 | 0.114 | 0.094 | 0.154 | 0.099 | 0.102 | 0.143 | 0.127 |
| HOL |  |  |  |  |  |  |  |  |  | 0.061 | 0.131 | 0.105 | 0.091 | 0.079 | 0.050 | 0.123 | 0.074 | 0.018 | 0.118 | 0.089 |
| IR |  |  |  |  |  |  |  |  |  |  | 0.119 | 0.085 | 0.049 | 0.051 | 0.017 | 0.116 | 0.036 | 0.054 | 0.096 | 0.057 |
| JER |  |  |  |  |  |  |  |  |  |  |  | 0.136 | 0.131 | 0.132 | 0.118 | 0.168 | 0.120 | 0.125 | 0.163 | 0.129 |
| MON |  |  |  |  |  |  |  |  |  |  |  |  | 0.109 | 0.099 | 0.083 | 0.143 | 0.097 | 0.097 | 0.137 | 0.064 |
| MRY |  |  |  |  |  |  |  |  |  |  |  |  |  | 0.091 | 0.036 | 0.123 | 0.014 | 0.082 | 0.122 | 0.096 |
| NRC |  |  |  |  |  |  |  |  |  |  |  |  |  |  | 0.049 | 0.127 | 0.076 | 0.072 | 0.092 | 0.072 |
| PRP |  |  |  |  |  |  |  |  |  |  |  |  |  |  |  | 0.113 | 0.018 | 0.039 | 0.097 | 0.055 |
| RDM70 |  |  |  |  |  |  |  |  |  |  |  |  |  |  |  |  | 0.112 | 0.117 | 0.151 | 0.139 |
| RDN |  |  |  |  |  |  |  |  |  |  |  |  |  |  |  |  |  | 0.053 | 0.088 | 0.058 |
| RH |  |  |  |  |  |  |  |  |  |  |  |  |  |  |  |  |  |  | 0.110 | 0.082 |
| SHO |  |  |  |  |  |  |  |  |  |  |  |  |  |  |  |  |  |  |  | 0.118 |
